# DNA barcodes from over-a-century-old type specimens shed light on the taxonomy of a group of rare butterflies (Lepidoptera: Nymphalidae: Calinaginae)

**DOI:** 10.1371/journal.pone.0305825

**Published:** 2024-07-17

**Authors:** Valentina Todisco, Dipendra Nath Basu, Sean W. J. Prosser, Stephen Russell, Marko Mutanen, Alberto Zilli, Blanca Huertas, Krushnamegh Kunte, Richard Vane-Wright

**Affiliations:** 1 Department of Environment and Biodiversity, Paris Lodron University of Salzburg, Salzburg, Austria; 2 National Centre for Biological Sciences, Tata Institute of Fundamental Research, Bengaluru, India; 3 University of Guelph, Guelph, Ontario, Canada; 4 Sciences Department, Natural History Museum, London, United Kingdom; 5 Ecology and Genetics Research Unit, University of Oulu, Oulu, Finland; Oxford Brookes University, UNITED KINGDOM

## Abstract

We analyzed COI barcode sequences from 138 over-a-century old specimens of *Calinaga* including 36 name-bearing type specimens stored at the Natural History Museum London. These new data, combined with previously available RPS5 sequences, divide the *Calinaga* samples into four well-supported mitochondrial lineages that together with a novel wing-pattern analysis, support the recognition of six species (*lhatso*, *buddha*, *brahma*, *aborica*, *formosana* and *davidis*), with all other names subsumed either as subspecies or synonyms. One new taxon is described, *Calinaga aborica naima* Vane-Wright, **ssp. n**.

## Introduction

Natural history collections have long been used by morphologists and taxonomists to probe the evolutionary process and describe biological diversity. They are often critical when it comes to investigating the taxonomy and evolutionary history of species that today are rare or extinct, undergoing particular conservation measures, or are simply difficult or costly to sample or collect in the field [1]. Furthermore, the preservation of type specimens represents an invaluable source of information for those taxa in need of taxonomic revision. However, samples from museum specimens tend to be difficult to work with because of their age.

Recent advances in DNA methodology, notably high-throughput sequencing (HTS), are fundamentally revolutionizing the acquisition of genetic data, including information from historical specimens [2–6], making genomic sampling from museum specimens more efficient. *Calinaga* Moore, [1858] forms a monotypic subfamily of butterflies distributed from Indochina to Taiwan and the Himalayas. Depending on the source consulted, *Calinaga* is composed of anywhere from one to eleven species [7–11]. In addition, due to its unique morphological characteristics and previously uncertain position in the Nymphalidae family tree, the genus has been subject to broad scholarly curiosity [12–23].

A modern large-scale study employing molecular and morphological characters from wing pattern and genitalia of nearly all species (except *C*. *formosana* Fruhstorfer [24]) addressed some of these long-standing issues, highlighting the presence of four main lineages within *Calinaga* (*C*. *aborica*, *C*. *davidis*, *C*. *lhatso* and *C*. *sudassana*). However, a recent review of the genus [25] has introduced a unique taxonomic scheme for the genus, dividing it into five “superspecies” (*buddha*, *brahma*, *formosana*, *davidis* and *lhatso*), with extreme over-splitting of the populations into many semi-species and sub-species based on inconsistent and weak wing-pattern elements, adding new confusion to the taxonomy in this group.

Recognizing the persistent problems surrounding the taxonomic status of the named taxa within *Calinaga* and the species boundaries within the genus, we here re-address the issue using a novel morphological approach to wing-pattern analysis as well as mitochondrial COI barcodes, and existing Ribosomal Protein S5 gene (RPS5) data. Most of this information has been gathered from the majority of taxa available in what is believed to be the largest world collection of *Calinaga* butterflies (>2000 specimens), hosted at the Natural History Museum of London (NHMUK). In the process we obtained, for the first time, COI barcode sequences from many of the original +100-year-old type specimens of *Calinaga*. Given the taxonomic coverage in our datasets, we aimed to provide dramatic new insights into the taxonomy of *Calinaga*.

## Materials and methods

### Taxon sampling

We sampled legs from 138 *Calinaga* individuals stored in NHMUK for DNA analysis, including all type specimens available for sampling (N = 36). All specimens were photographed using a Canon Eos 600D with 70mm Sigma DG Macro lens at the Museum using standard photography equipment. The non-type material was initially identified by RIVW and included representatives of 15 taxa: *aborica*, *buddha*, *brahma*, *buphonas*, *formosana*, *bedoci*, *gautama*, *cercyon*, *davidis*, *funebris*, *funeralis*, *lhatso*, *sudassana*, *senseiensis* and *pacifica*. Samples were submitted for sequencing using the original identifications but were later re-identified according to Tshikolovets [25] subsequent to collection of genetic and re-analysis of morphological data (see paragraph 2.5) (S1 Table). A few mysterious (i.e. *dubernardi* Oberthür, 1920) or recently described taxa (i.e. *yaonica* Sugiyama, 2015; *hsui* Tshikolovets, 2020; *songyunlangi* Tshikolovets, 2020) could not be sampled.

### DNA extraction

A single leg was removed from each *Calinaga* specimen and the extraction of mtDNA was conducted in two different laboratories, the NHMUK, and the Ecology and Genetics Research Unit (EGRU, University of Oulu, Oulu, Finland). Total genomic DNA was extracted from one leg of each individual using the Qiagen columns of QIAamp DNA mini kit and DNeasy Blood & Tissue Kits (Qiagen, Hilden, Germany) in NHMUK lab and EGRU lab respectively and it was eluted in 20–50μl of Elution buffer. The eluted DNA obtained in EGRU lab was sent to the Canadian Centre for DNA Barcoding (CCDB) (University of Guelph, Guelph, Canada) for sequencing.

### High-throughput and Sanger sequencing

Due to DNA degradation in the very old specimens of *Calinaga* in CCDB, the 658 bp COI barcode region was recovered using an HTS-based protocol [26]. In brief, for each sample, multiple short, overlapping amplicons were generated using nested, multiplex PCR. In order to associate reads with their source specimen, the amplicons were tailed with sample-specific universal molecular identifiers (UMI) before being pooled for sequencing on an Ion Torrent PGM. The short sequence reads were attributed to a sample via the UMIs, and filtered for quality and length before being assembled into a single barcode sequence. For the remaining specimens, PCR and Sanger sequencing were carried out in NHMUK lab. Briefly, the 658 bp barcode region was amplified using the primers described in Prosser et al. [26] as well as two forward and two reverse primers for *Calinaga* designed by VT (S1 File) and sequenced on an ABI 3730XL (Applied Biosystems capillary sequencer). The trace files were edited using CodonCode Aligner 6.0.2 (CodonCode Corporation, Dedham, Massachusetts) and all resulting mtDNA sequences were aligned using the same program. All sequences were submitted to GenBank (accessions PP595598-PP595669, see S1 Table) and BOLD system repository (dataset “DS-CALINHM”, accessible at https://dx.doi.org/10.5883/DS-CALINHM).

### Data set compilation and tree reconstruction procedures

To assess the phylogeographic patterns within *Calinaga*, we complemented the 138 sequences obtained through this study with an additional 51 mtDNA and 24 RpS5 (ribosomal protein S5 nuclear gene) sequences from Todisco et al. [24]. Another five publicly available mtDNA sequences of *Calinaga* were retrieved from GenBank and added to the dataset. In addition, mtDNA sequences for six species belonging to closely related lineages of Nymphalidae (Charaxinae and Satyrinae) from Wahlberg and Wheat [20] were retrieved from GenBank and added to our dataset as outgroups (S1 Table).

All sequences were aligned using CodonCode Aligner 6.0.2. The mean p-distance between and within the main haplogroups of mtDNA sequences and its variance (bootstrap method, 500 replicates) was calculated using MEGA 11 [27] (Table 1).

**Table 1:** Average uncorrected p-distances (in % of the COI barcoding region) and standard deviation between and within groups.

	**Ia**	**Ib**	**Ic**	**II**	**III**	**IV**
**Ia. *lhatso***	0.5±0.4					
**Ib. *buddha***	1.3±0.4	0.6±0.6				
**Ic. *brahma***	1.5±0.3	0.8±0.3	0.2±0.2			
**II. *aborica***	5.5±0.6	5.1±0.6	5.5±0.4	0.3±0.2		
**III. *formosana***	6.3±0.7	6.4±1.1	6.2±0.8	4.4±0.5	0.2±0.1	
**IV. *davidis***	7.4±1.4	7.3±1.1	7.4±0.8	5.7±0.6	2.3±0.4	0.6±0.5

The mtDNA and RpS5 sequences were concatenated and IQ-TREE 1.6.11 [28] was used to select the best fitting model of evolution by Bayesian information criterion scores (BIC) and the Maximum Likelihood (ML) analysis was carried out with the same program. Bayesian analysis was allowed to run with the same dataset in BEAST 2.7.3 [29] for 20 million generations and was repeated multiple times to check for convergence and stationarity, and the results were tested using TRACER 1.7.1 [30]. The resulting consensus tree was viewed in FigTree 1.4.4 [31].

### Wing colour pattern analysis

Photos of 156 NHMUK specimens from 16 *Calinaga* taxa (listed in S1 Table) were quantified for FW and HW colour-pattern. Most taxa had dark background and interspersed light colour patches on both wings, therefore we chose the position of dark colour on wings to extract wing colour patterns. Wing colour patterns were extracted, aligned, and analyzed using the R package ‘patternize’ [32]. Within taxon variation in the pattern were obtained and visualized as heatmaps by summing the separate binary raster stacks of forewing and hindwing using *sumRaster* function [32]. Proportion of background pattern to the total wing area for each taxon was then calculated using the *patArea* function for both FW and HW [32], and PCA was performed on the transformed binary dataset obtained from the raster of each sample. To understand the extent of difference between mean colour patterns we used the PCs to calculate the Euclidean distances and estimated significance using randomized residual permutation procedure [33] (Table 2). Euclidean distance, relative area calculations, and heatmap visualization were calculated only for taxa for which we had at least 5 samples.

**Table 2:** Euclidean distances between wing colour patterns of 11 taxa. In the matrix lower and upper half represents forewing and hindwing, respectively. Significant distances are marked in bold. The significance of the distances was based on a MANOVA associated randomized residual permutation procedure.

**Taxa**	*avalokita*	*brahma*	*buddha*	*buphonas*	*davidis*	*funebris*	*gautama*	*lactoris*	*lhatso*	*naima*	*sudassana*	Hindwing
*avalokita*	NULL	81.525	86.733	99.091	94.763	111.219	86.240	113.150	98.501	92.355	87.526	
*brahma*	85.558	NULL	64.725	77.103	61.300	92.872	80.545	106.226	95.075	72.114	78.883	
*buddha*	115.346	84.973	NULL	85.681	76.187	91.727	92.976	106.201	94.129	80.859	89.265	
*buphonas*	122.710	100.768	81.020	NULL	80.058	94.150	90.962	101.605	100.331	83.425	88.868	
*davidis*	113.781	89.430	73.619	80.679	NULL	95.876	86.690	97.909	94.490	80.101	84.543	
*funebris*	128.535	102.280	91.991	85.471	81.598	NULL	103.507	89.627	96.915	83.135	106.121	
*gautama*	125.601	99.866	97.184	86.180	84.854	85.922	NULL	101.339	90.401	79.540	72.114	
*lactoris*	123.038	105.865	101.945	96.954	91.262	113.671	99.487	NULL	87.601	83.083	104.727	
*lhatso*	131.819	105.732	83.651	103.605	86.054	108.345	115.366	118.125	NULL	81.182	90.462	
*naima*	129.669	104.473	86.471	93.068	80.198	98.325	94.382	114.914	86.674	NULL	78.463	
*sudassana*	116.359	83.671	55.992	94.095	83.561	101.048	104.256	106.253	85.976	93.818	NULL	
Forewing												

We prepared a morphological key for *Calinaga* species-groups according to the findings of this study. In addition, we conducted an Elliptical Fourier transformation analysis on the wing outlines as well as a landmark-based analysis for wing venation. These analyses also showed some taxonomic information; they are included as supporting information (S2–S4 Files).

### Nomenclatural acts

The electronic edition of this article conforms to the requirements of the amended International Code of Zoological Nomenclature, and hence the new names contained herein are available under that Code from the electronic edition of this article. This published work and the nomenclatural acts it contains have been registered in ZooBank, the online registration system for the ICZN. The ZooBank LSIDs (Life Science Identifiers) can be resolved and the associated information viewed through any standard web browser by appending the LSID to the prefix ""http://zoobank.org/"". The LSID for this publication is: urn:lsid:zoobank.org:pub:1BA7E8E9-8C85-4799-A664-9E6911DB97DF. The electronic edition of this work was published in a journal with an ISSN, and has been archived and is available from the following digital repositories: PubMed Central, LOCKSS [author to insert any additional repositories].

## Results

### Sequencing results

COI barcode sequences of various lengths (S1 Table) were recovered for sixty-four out seventy-one (HTS) and twelve out of sixty-eight (Sanger sequencing) samples respectively. These sequences matched well with other *Calinaga* barcodes in BOLD Identification System. Further confirmation of their validity was provided by phylogenetic methods and by the fact that they grouped with sequences from closely allied taxa. Failure to obtain the full-length (658 bp) barcode sequences for all samples can be attributed to amplification failure of the COI gene, in particular in Sanger sequencing method, due to DNA degradation. In addition, the identity of GenBank sequence ON437174 (*C*. *sudassana*, misidentified as *C*. *buddha*) was double-checked and corrected in our dataset (D. Lohman, pers. comm.).

### Phylogenetic analysis

**Fig 1:**
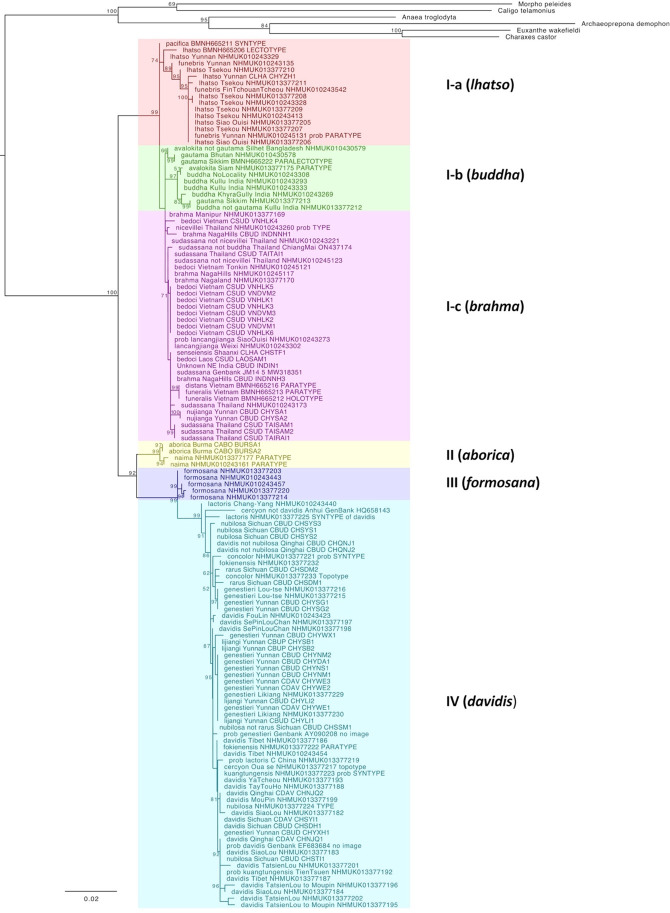
Maximum Likelihood phylogeny for *Calinaga* of combined data (COI+RpS5) inferred using IQTree. Only node support values over 50 are shown.

**Fig 2:**
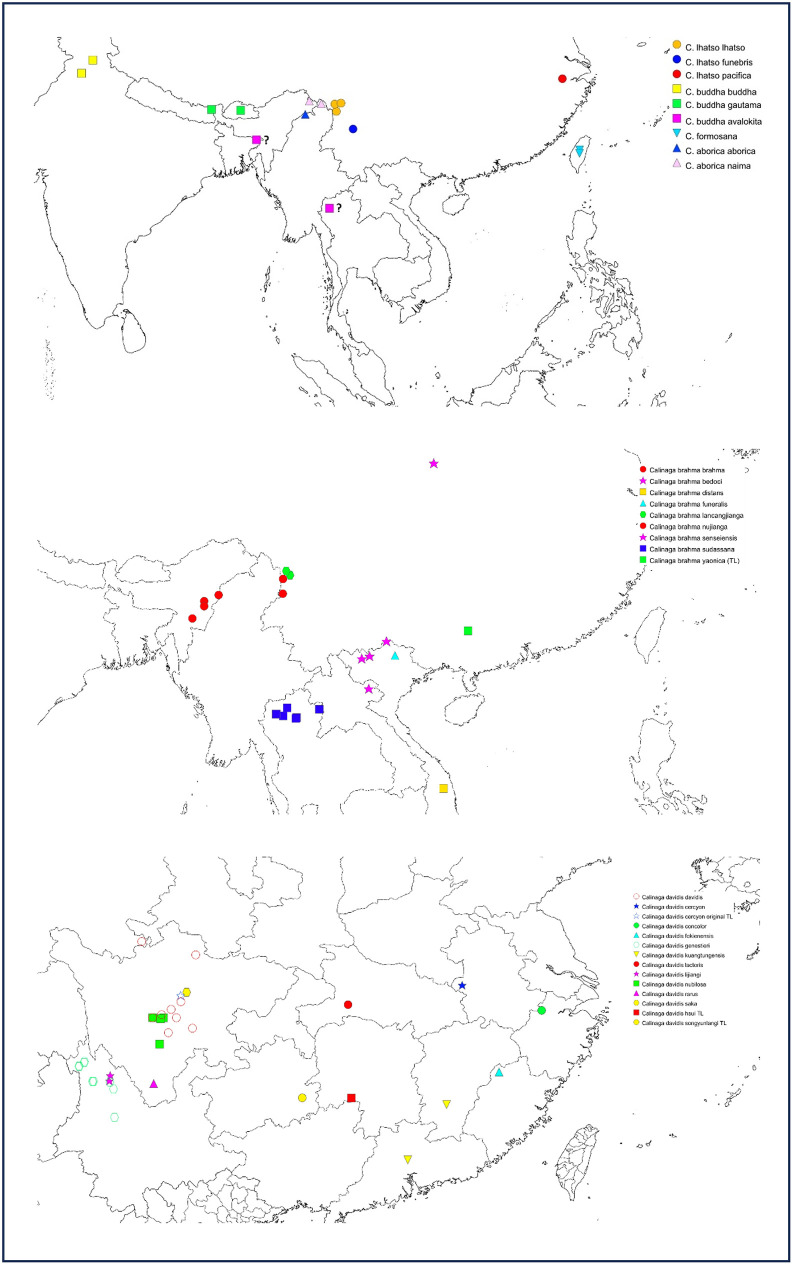
Distribution of the sequenced *Calinaga* specimens according to the taxonomy proposed in this study. a) *C*. *lhatso*, *C*. *buddha*, *C*. *formosana* and *C*. *aborica*, b) *C*. *brahma*, and c) *C*. *davidis*. Locations for specimens of *C*. *buddha avalokita* are uncertain (see Results). Map generated using Simplemappr.net (in public domain, https://www.simplemappr.net/).

Our Maximum Likelihood (Fig 1) revealed four well-supported clades corresponding to: I) *lhatso + brahma + buddha*, II) *aborica*, III) *formosana*, and IV) *davidis* groups. Within the first group, three shallow subclades (Ia, Ib and Ic), even though weakly supported, consistently appeared together throughout the analyses. In the subclade Ib the *C*. *buddha avalokita* specimens sequenced in this work carry location labels of Silhet (now in Bangladesh) and Thailand (Fig 2). Both these are doubtful since Silhet does not have the elevation and habitat suitable for *Calinaga*, and the Thailand label lacks details. Thus, the true location of *avalokita* is uncertain. The specimens were collected perhaps in Meghalaya, Patkai Hills or nearby ranges, and brought to the British outpost in Silhet. Our phylogeny showed the following taxonomic incongruencies with the recent classification of Tshikolovets [25]: **a)** the taxon *funebris* appeared as part of the larger variation within the *lhatso* group and not as a separate lineage; **b)** taxa *funeralis* and *senseiensis* appeared as part of the larger variation within *brahma* group and not as a separate lineages; **c)** taxon “*naima”* (here described) showed unique genetic characteristics distinct from *aborica*, **d)** our only sequenced sample of the taxon *pacifica* appeared very close (and as a sister) to the *lhatso* group.

Our BEAST tree (S5 File) showed a similar topology to the ML tree and therefore will not be discussed further.

### Colour patterns

**Fig 3:**
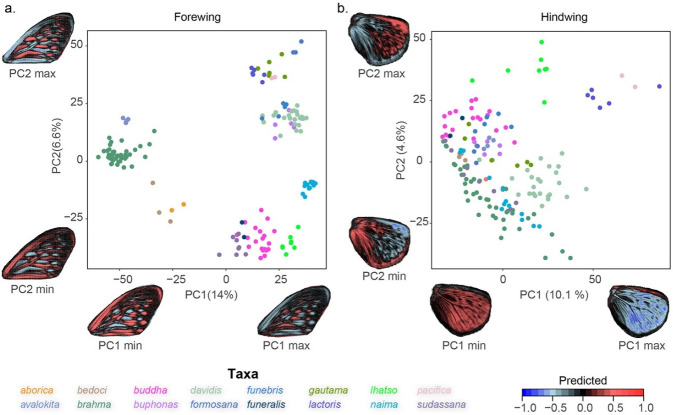
PCA on raster stacks of dark background colour pattern in forewing (a) and hindwing (b). Predicted colour pattern changes along first two PC axes are also shown. In the cartoon, positive values presenting a higher predicted presence of the specific pattern are in red and negative values presenting the absence of the pattern are in blues.

**Fig 4:**
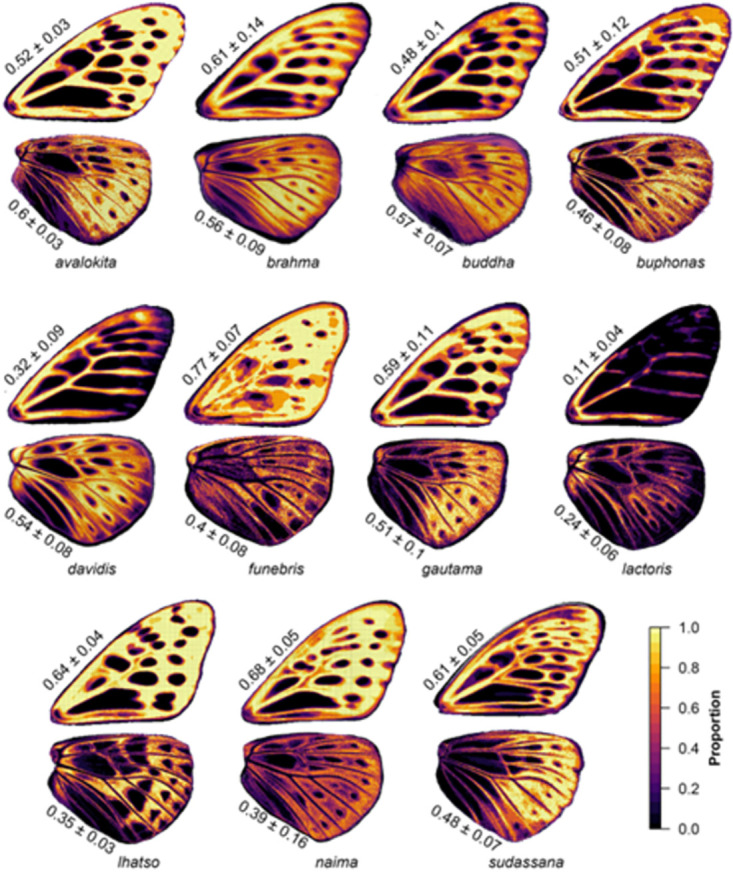
Quantification of dark background colour pattern in 11 taxa. Heatmaps demonstrate the consistency of colour pattern yellow indicating consistent presence of patterns and red-dark purple gradient indicating less consistent presence. The black areas represent the lighter interspersed patches. Mean and standard deviation of relative size (proportion of the wing) of background colour pattern are also shown.

We extracted the dark background surrounding the paler patches delineating wing patterns. In the PCA on forewing colour patterns, the first two axes showed changes in position of lighter patches (Fig 3a). In hindwing the first PC axes showed presence or absence of a particular colour pattern phenotype rather than change in spatial distribution of interspersed lighter patches, which is shown along PC2 (Fig 3b). In the morphospace, we observed a more constrained forewing colour pattern compared to variable and overlapping hindwing colour pattern across taxa. Pairwise MANOVA showed significant differences in mean colour pattern between almost all taxa pairs for both forewing and hindwing (Table 2). Intra-taxon variation was visualized as heatmaps, along with relative area of the dark background on the wings of each taxon (Fig 4). These results showed that in FW, *davidis* and *lactoris* have relatively less dark background area along with larger light patches, which is distinct from other taxa, while in hindwings, *lactoris* and *lhatso* have similar patterns.

## Discussion

Although many studies show the importance of combining different markers to obtain robust species-level identification [34], COI barcode sequences have proven to be sufficient in differentiating species in a wide range of organisms [35]. In this study our morphological analyses in conjunction with COI and RPS5 sequence data shed light on the long-standing problem of the systematics in genus *Calinaga*.

Despite evidently being a geologically old genus [24, 36, 37], *Calinaga* does not demonstrate nearly the same degree of species divergence and differentiation that is prevalent in other nymphalid groups of similar age. This pattern is typical of “living fossils”, ancient lineages with one or few living representatives, such as the Mexican *Baronia brevicornis*, the lone species in a monotypic subfamily that is sister to all other swallowtails [38]. Similarly, the limited morphological differentiation in wing pattern, combined with individual variation and clinal gradients, has been the main source of historical confusion about the systematics of *Calinaga*.

### Taxonomic consideration

Ever since its 19^th^ century description by Frederic Moore, there has been much taxonomic uncertainty regarding not only the higher classification of *Calinaga* within the butterflies, but also, as the diversity of named phenotypes included in the genus increased, the relative, species-level status of its component taxa. Ehrlich [7, 18] effectively anticipated the solution to the higher classification problem (the Calinaginae are now convincingly regarded as the sister group of the remainder of the satyroid clade, within Nymphalidae *sensu lato* [36, 37]), but Ehrlich was non-committal on the species diversity issue: He simply treated *Calinaga* as a single, polytypic species. Ever since, the number of *Calinaga* species to be recognised has remained very uncertain.

In his recent monographic account, Tshikolovets [25] divided *Calinaga* into five superspecies: *buddha*, *brahma*, *formosana*, *davidis* and *lhatso*–and among these, a total of 16 named species and semispecies (some with several subspecies).

At superspecies level, Tshikolovets’s classification is largely congruent with our results, but with some exceptions. We found the following groups:

#### I-a. *Calinaga lhatso*

Under this group, Tshikolovets lists *lhatso*, *senseiensis*, *pacifica*, *dubernardi*, *funebris* and *funeralis* as valid “semi-species”. Our results show that taxa *lhatso*, *pacifica*, and *funebris* undoubtedly belong to this clade. The oldest name in this clade is *lhatso* Oberthür, 1893 and therefore we recognize this clade as *Calinaga lhatso* with subspecies *lhatso* and *funebris* **stat. nov**. Our single specimen of *pacifica* appeared as sister to the remaining samples in this clade and may perhaps later prove to be a “good” species; however for now we consider it part of the variation in this clade. The little-known taxon *dubernardi* Oberthür, 1920 (TL: “Tsekou” [Cigu, Deqin, Yunnan]), provisionally included in this group, has no surviving type material, but according to the original description it shows intermediate characteristics between *lhatso* and *brahma*; its type locality is the same as *lhatso* and it is possibly a form of it or a hybrid between *lhatso* and *brahma* [25]. In our phylogenetic analysis, the taxa *senseiensis* (one specimen) and *funeralis* (holotype and one paratype) did not appear to belong to this group but to *brahma*; however for now we maintain their position in the *lhatso* group as *incertae sedis* until further evidence to support their position becomes available. We could not sample the taxon *yaonica*, but morphologically it appears to belong to this group and be closely related to *funeralis*.

#### I-b. *Calinaga buddha*

Our results largely support the taxonomy proposed by Tshikolovets [25] for this group, who recognized three taxa within it: *buddha*, *avalokita* and *gautama*. The *C*. *buddha avalokita* specimens sequenced in this work carry location labels of Silhet (now in Bangladesh) and Thailand. Both these are doubtful since Silhet does not have the elevation and habitat suitable for *Calinaga*, and the supposed Thailand source lacks details. Thus, the true location of *avalokita* is uncertain. The specimens were collected perhaps in Meghalaya, Patkai Hills or nearby ranges, and brought to the British outpost in Silhet. Even though in our results this group is not monophyletic, the samples always cluster together in a closed group. Since no geographic distinction could be inferred from our phylogeny supporting separation of these taxa at species level, we recognize *Calinaga buddha* with three subspecies: *buddha*, *avalokita* and *gautama* **stat. rev**. The placement of *gautama* as a subspecies of *C*. *buddha* is tentative and requires further taxonomic study.

#### I-c. *Calinaga brahma*

Tshikolovets [25] recognized *sudassana*, *nicevillei*, *bedoci*, and *distans* under *brahma* as well as two new subspecies (*nujianga* and *lancangjianga*). Our results confirm that these taxa do indeed fall within the *brahma* clade. Even though DNA barcodes do not discriminate between any of these taxa, they all appear to be allopatric in distribution and potentially represent valid subspecies (Fig 2). We therefore recognize them as subspecies of *brahma* (but see above for taxa *senseiensis* and *funeralis*).

#### II. *Calinaga aborica*

Todisco et al. [24] found *aborica* to be a distinct and well-supported separate species, which was further confirmed by a subsequent, independent analysis [36]. Tshikolovets [25] nevertheless listed *aborica* as a semi-species under superspecies *davidis*. Our results yet again support the recognition of *aborica* (TL: “Abor Hills” [Arunachal Pradesh, NE India]) as a separate species very distinct (>5%) from the members of the *davidis* group, with the geographically separated populations from Assam and N Burma (Kachin) as a separate subspecies. These populations were named “*naima*” by Vane-Wright in 1971 (*in litt*., nomen nudum) [39]. Here we formally describe this taxon for the first time (for a complete synopsis of *Calinaga aborica*, see S3 File).

**Fig 5:**
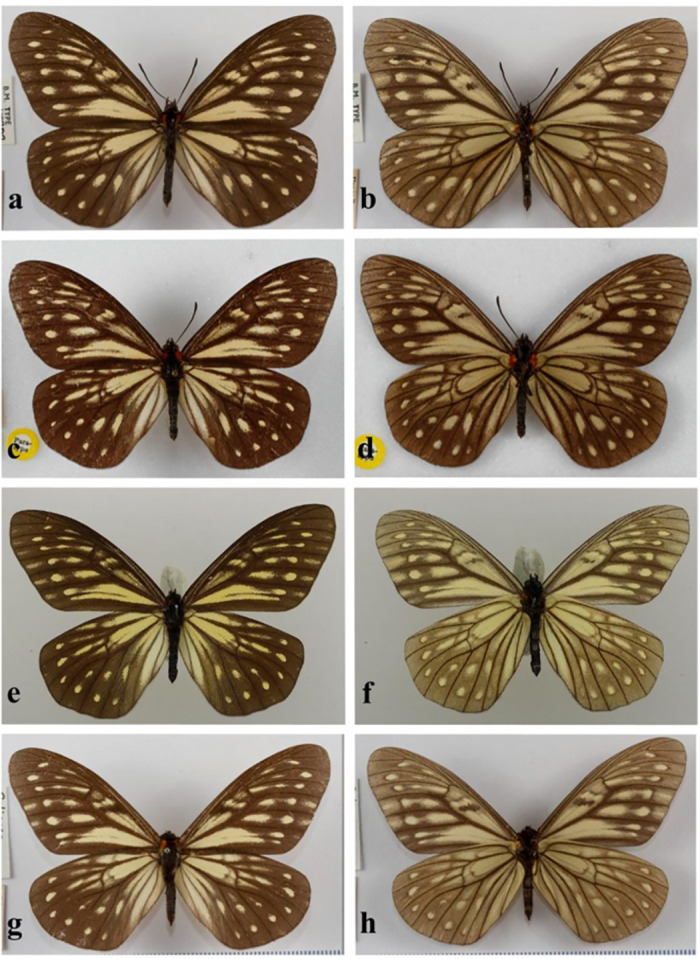
*Calinaga aborica naima* Vane-Wright, ssp. nov. a,b: holotype, c–h: paratypes, all males, uppersides left, undersides right (a–d, g,h NHMUK; e,f Sukkit Collection). For additional data see S3 File.

***Calinaga aborica naima*** Vane-Wright **ssp new** (Fig 5)

*Calinaga aborica naima* Vane-Wright ssp. nov. urn:lsid:zoobank.org:act:773B49ED-C54E-4D76-8E61-267F5722320C

Barcode Index Number: BOLD:ACZ3798

#### Diagnosis

**Fig 6:**
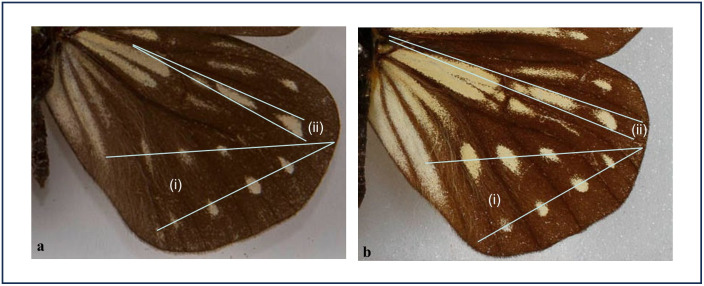
a) *Calinaga aborica aborica* (Lectotype). (i) Divergent centres of submarginal and postdiscal spots in HW cells M_3_, CuA_1_ and CuA_2_. (ii) Anterior and posterior tangential lines touching the main postdiscal streak and submarginal spot in HW cell M_1_ converge well before the base of the wing. b) *Calinaga aborica naima* (paratype 010243161). (i) Divergent centres of submarginal and postdiscal spots in HW cells M_3_, CuA_1_ and CuA_2_. (ii) Anterior and posterior tangential lines touching the main postdiscal streak and submarginal spot in HW cell M_1_, even if converging, do not converge before the base of the wing. Note also how, in subsp. *naima*, the yellow scales in HW cells 1A and 2A are more dense, and extend almost to the wing margin.

This new subspecies shares at least three morphological characters with typical *Calinaga aborica* that link the two taxa together (Clade II), while separating them from all other known *Calinaga*: red hairs on the thoracic dorsum restricted to the tegulae, hindwing discocellular veins *m1–m2* and *m2–m3* form a slight but distinct obtuse angle (*ca* 150–160°), and postdiscal and submarginal pale spots in hindwing cells M3, CuA1 and CuA2 uniquely arranged (Fig 6a and 6b(i); see S3 File for more information). Subspecies *naima* differs from the nominotypical, western subspecies in generally having a much ‘brighter’ wing-pattern–although, as with subsp. *aborica*, there is considerable individual variation in all pale markings. Two colour pattern features are considered diagnostic:

- In subspecies *naima*, on the underside, the dark ground colour is relieved by numerous yellow scales, these being dense in many areas, including the bases of hindwing cells CuA_1_ and CuA_2_. In contrast, these yellow scales in subsp. *aborica* are far less dense, including those at the bases of hindwing cells CuA_1_ and CuA_2_, which areas are thus far darker.
- The subtriangular, submarginal pale spot in cell M_1_ on the hindwing upperside varies from being slightly wider than, to about the same width as the postdiscal streak in the same cell, such that if virtual anterior and posterior tangential lines are drawn to touch these two marks, the lines do not converge before the base of the wing, being more or less parallel (Fig 6b(ii)). In subsp. *aborica* such virtual lines converge before the base of the wing (Fig 6a(ii)).

Typically in subsp. *naima* (Fig 5), in comparison to subsp. *aborica*, the pale postdiscal streaks in forewing upperside cells R_5_, M_1_ and M_2_ are slightly better developed (streak in cell R_5_ *ca* 3–4 mm); the pale streaks in hindwing upperside cell R_1_ are generally far more prominent; the distal area of hindwing upperside cells 2A and 3A are often pale yellow almost to the margin (Fig 6b, cf. Fig 6a); and on the hindwing underside, the distal triangular spot in cell R_1_ is always present, usually larger and less dyslegnic (i.e., having its margins more sharply defined), about 2.5–5.5 mm in length.

**Forewing length (male)**: mean 40.96 mm [n = 17; observed range 37.6–43.1 mm, SD 1.441]; female unknown.

**Material directly examined**: holotype and 12 paratypes in NHMUK, London

**Photographs examined**: 3 paratypes in the collection of the late Prasobsuk Sukkit (courtesy of Adam Cotton, Chiang Mai) and 1 paratype, ex Sukkit collection, in the collection of Howard Grisham, Alabama. Another specimen ex Sukkit collection, a specimen from ‘Wanzewong-Ngawar’ figured by Shizuya et al. [40]; and an online image of a male from ‘Chudu Razi Hills’ [see S3 File], are not included as paratypes.

#### Holotype ♂

**Myanmar** / Upper Burma: Seinghku Valley, 6500′, 28.5 N 97.35 E., 27.v.1926, F. Kingdon Ward. / Brit. Mus. 1926–400 / BMNH(E)#985060 / B.M. Type No. Rh. 17202 / *Calinaga aborica naima* Vane-Wright Holotype ♂ det. R.I. Vane-Wright 1971 / NHMUK. [See S3 File on Kingdon Ward’s localities.]

#### Paratypes

**Myanmar**–1 ♂ / Upper Burma: Seinghku R.[iver], 28.3 N, 97.31 E, 5000′, 17.v.1926, F. Kingdon Ward [see Kingdon Ward, 1930] / NHMUK. 3♂♂ / N. Burma: Adung Valley, 6000′, 14.v.1931, Lord Cranbrook [one specimen barcoded] / NHMUK. 2♂♂ / Mairudam, N Kachin State, Burma Prasobsuk Sukkit leg. 5 May 1998 / 5.v.1998 N.E. Putao. Kachin State Myanma [sic] Sukkit & Nishimura Coll. / 1♂ / Mairudam, N Kachin State, Burma Prasobsuk Sukkit leg. 14 May 1998 / 14.v.1998 N.E. Putao. Kachin State Myanma [sic] / Sukkit & Nishimura Coll. 1♂ / Mairudam, 12.v.1998 N.E. Putao. Kachin State Myanma [sic] leg. Sukkit & Nishimura / Howard Grisham Coll.

**India**–3♂♂ Assam, Mishmi Hills, 1928, Percy Sladen Expn. / 2♂♂, Assam, Mishmi Hills, 2000′, 3.iii.1928, Percy Sladen Expn. / 28°0′ N. 96°0′ E / [one specimen barcoded] /, 1♂, Assam, Mishmi Hills, 4500 ft, 11.v.1928, Percy Sladen Expn. / Delei Valley [see Kingdon Ward, 1930], 28°21′ N, 96°37′ E, 4500ʹ, 11/5/28. / 2♂♂, Assam, Mishmi Hills, 10000 ft, 13.vi.1928, Percy Sladen Expn. / 28°21′ N, 96°37′ E /. [all NHMUK]

**Distribution**: **India****–Arunachal Pradesh**: Mishmi Hills. **Myanmar****–N Kachin State**: Seinghku Wang Valley, Adung Valley, Mairudam (NE Putao), Wanzewong-Ngawar (north of Putao), Chudu Razi Hills (*ca* 40 km east of Kawnglangphu). [For details, including estimated coordinates, see S3 File.]

**Flight period and altitudinal range**: records are for March–June; altitude 600–3000 m (mid-montane).

#### Etymology and nomenclature

Named after the ballad ‘Naima’, originally composed and performed by saxophonist John Coltrane for his 1960 album *Giant Steps*. The name was chosen (and the NHMUK specimens so-labelled) in 1971, but it has not been formally established until now. The name *naima* has, however, appeared in the literature at least three times as a *nomen nudum* or manuscript name [11, 25, 39]. While it is sometimes best to avoid subsequent establishment of a *nomen nudum*, in this case little purpose would be served by introducing a novel alternative; *Calinaga aborica naima* is here established with Vane-Wright (in Todisco et al. [24]) as author, and should be dated from the publication date of this paper (ICZN [41]: glossary).

#### III. *Calinaga formosana*

This isolated taxon from Taiwan, which was not included in the analysis of Todisco et al.’s [24], appears as a well-supported, distinct clade. We confirm its undisputed status as a valid species (see also S4 File), with no apparent sub-population structure.

#### IV. *Calinaga davidis*

This complex includes several poorly-characterized taxa that are very difficult to differentiate objectively. Beside *C*. *aborica* which, based on our findings, is undoubtedly a separate species outside of the *davidis* group, Tshikolovets [25] listed three “semi-species” (*davidis*, *cercyon* and *buphonas*), each with four or five subspecies. While we could not sample three of the described taxa (i.e. *saka*, *hsui* and *songyunlangi*), we can confirm that all the others listed by Tshikolovets appear together in a large cluster without any particular distinction, many represented by geographically isolated allopatric populations that can be thought of as subspecies of a single species. However, the situation in central Sichuan, where taxa *davidis*, *nubilosa* and *saka* occur in part in sympatry, is particularly complex (Fig 2). We agree with the argument presented by Tshikolovets [25] that the original type locality of *cercyon* (“The road between Tâ-Tsien-Loû and Mou-Pin, and the neighborhood of Tâ-Tsien-Loû, Western China” [42] is incorrect, and the types probably originated from somewhere else.

We found specimens attributable to ssp. *lijiangi* flying together with specimens of *genestieri* in Northern Yunnan, sharing the same barcode (Fig 1). The taxon *lijiangi*, described based solely on the lighter wing coloration compared to *genestieri*, therefore appears to be synonymous with the latter (**syn. nov**.). In addition, the taxa *saka* and *nubilosa* fly sympatrically with *davidis* and are morphologically and genetically indistinguishable from one another. Here we synonymize these two with *davidis* (**syn. nov**.). Until further evidence is presented, here we maintain the status of the remaining taxa in the group as valid allopatric subspecies of *Calinaga davidis*.

We also present an illustrated, semi-natural key to the six subgroups of *Calinaga* detailed above (S4 File). These conclusions are further supported by our morphological analysis of wing pattern that unambiguously separates many of the recognized taxa into distinct clusters (Fig 1). Thus, we propose the following revised classification for *Calinaga*:

1) *Calinaga lhatso* Oberthür, 1893
  a. ssp. *lhatso* Oberthür, 1893
  b. ssp. *funebris* Oberthür, 1919
  c.? ssp. *funeralis* Monastyrskii and Devyatkin, 2000 *incertae sedis*
  d.? ssp. *senseiensis* Yoshino, 1997 *incertae sedis*
  e.? ssp. *dubernardi* Oberthür, 1920 *incertae sedis* (not examined)
  f.? ssp. *yaonica* Sugiyama, 2015 *incertae sedis* (not examined)
  g.? ssp. *pacifica* Mell, 1939 *incertae sedis* (possibly a separate species)
2) *Calinaga buddha* Moore, [1858]
  a. ssp. *buddha* Moore, [1858]
  b. ssp. *gautama* Moore, 1901
  c. ssp. *avalokita* Fruhstorfer, 1913
3) *Calinaga brahma* Butler, 1885
  a. ssp. *brahma* Butler, 1885
  b. ssp. *sudassana* Melvill, 1893
  c. ssp. *nicevillei* Oberthür, 1920
  d. ssp. *bedoci* Le Cerf, 1926
  e. ssp. *distans* Monastyrskii and Devyatkin, 2000
  f. ssp. *lancangjianga* Tshikolovets 2020
  g. ssp. *nujianga* Tshikolovets 2020
4) *Calinaga aborica* Tytler, 1915 **stat. rev**.
  a. ssp. *aborica* Tytler, 1915
  b. ssp. *naima* Vane-Wright, 2024 **ssp. nov**.
5) *Calinaga formosana* Fruhstorfer, 1908
6) *Calinaga davidis* Oberthür, 1881
    = *saka* Moore, 1901 **syn. nov**.
    = *nubilosa* Oberthür, 1920 **syn. nov**.
  a. ssp. *davidis* Oberthür, 1881
  b. ssp. *cercyon* de Nicéville, 1897
  c. ssp. *lactoris* Fruhstorfer, 1908
  d. ssp. *fokienensis* Fruhstorfer, 1914
  e. ssp. *buphonas* Oberthür, 1920
  f. ssp. *genestieri* Oberthür, 1922
    = *lijiangi* Tshikolovets, 2020 **syn. nov**.
  g. ssp. *kuangtungensis* Mell, 1952
  h. ssp. *concolor* Mell, 1952
  i. ssp. *rarus* Monastyrskii, 2012
  j.? ssp. *hsui* Tshikolovets, 2020 *incertae sedis* (not examined)
  k.? ssp. *songyunlangi* Tshikolovets, 2020 *incertae sedis* (not examined)

## Supporting information

S1 Table List of material examined for molecular data. (XLSX)

S1 File VT: Newly designed COI primers. (PDF)

S2 File DB & KK: Morphological analysis of wing patterns. (PDF)

S3 File RIVW: Morphotaxonomy of subfamily Calinaginae, with special reference to *Calinaga aborica*. (PDF)

S4 File RIVW: Illustrated, semi-natural key to the major subgroups of *Calinaga*. (PDF)

S5 File BEAST tree for *Calinaga* of combined data (COI+RpS5). (PDF)
