# Supplementary material for: DNA barcodes from over-a-century-old type specimens shed light on the taxonomy of a group of rare butterflies (Lepidoptera: Nymphalidae: Calinaginae)
Source: PLoS One. 2024 Jul 17;19(7):e0305825. doi: 10.1371/journal.pone.0305825 (PMC11253935; doi:10.1371/journal.pone.0305825)

Supplementary Information S2

New Primers designed for *Calinaga*
By Valentina Todisco (valentina.todisco@plus.ac.at)

Cali 1

5’AACTTTATAYTTTATTTTTGGAATTTGAGCYGG3’

Cali 2

5’TTCTYYTAATTTCAAGAAGAATTGTAGAAAATG3’

Cali 3

5’AAGATCCRCYATGRGCAATATTAGATGAAAGTG3’

Cali 4

5’AAATAAATGTTGRTAYAAAATAGGRTCTCCYCC3’

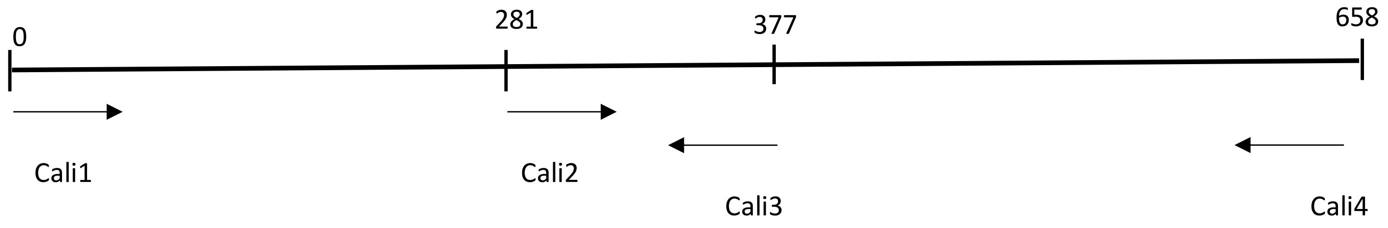

Supplement: S1 File — (PDF) [file pone.0305825.s002.pdf]
