# Supplementary material for: DNA barcodes from over-a-century-old type specimens shed light on the taxonomy of a group of rare butterflies (Lepidoptera: Nymphalidae: Calinaginae)
Source: PLoS One. 2024 Jul 17;19(7):e0305825. doi: 10.1371/journal.pone.0305825 (PMC11253935; doi:10.1371/journal.pone.0305825)
Supplement: S2 File — (PDF) [file pone.0305825.s003.pdf]

Supplementary Information S3

Geometric morphometry

By Dipendra Basu and Krushnamegh Kunte

Materials and Methods

Elliptical Fourier transformation on the wing outlines: We quantified wing shape and size (centroid size) characterizing outline contours by elliptical Fourier analysis (Kuhl and Giardina 1982; Ferson et al. 1985). In this method x and y coordinates of outline contours are first represented as a series of elliptical harmonics. These ellipses are then each defined as the sum of one sine and one cosine curves by decomposing a periodic continuous function implementing an extension of Fourier series. Fourier transform calculates four co-efficients for each harmonic which are eventually normalized to remove allometric variations (Kuhl and Giardina 1982). These normalized Fourier coefficients (EFDs) could be used as shape variables for further analyses.

We have photographed 156 specimens from 16 named taxa under *Calinaga*: 2 *aborica*, 5 *avalokita*, 3 *bedoci*, 43 *brahma*, 20 *buddha*, 5 *buphonas*, 25 *davidis*, 3 *formosana*, 5 *funebris*, 2 *funeralis*, 6 *gautama*, 6 *lactoris*, 8 *lhatso*, 13 *naima*, 2 *pacifica*, 8 *sudassana*. We photographed all these specimens in the Natural History Museum, UK. We used dorsal view of the right forewing and the ventral view of the left hindwing from the photographs of the specimens to avoid overlapping areas of forewing and hindwing in the pinned specimens. When necessary because of damage in the wings, we photographed opposite wings and mirrored it before digitizing wings. To obtain the outline contour, binarized wing images (black wings on white background) were used to acquire x, y coordinates of the wing outlines implementing R package '*Momocs*' (Bonhomme et al. 2014). The outlines were then centered, scaled, aligned, and finally stacked (Fig SX1 a, c). Then we calibrated the minimum number of harmonics needed to define the wing outlines. We found EFDs from seven harmonics for forewing and 10 harmonics for hindwing delineating the wing outlines with 99% accuracy (Fig SX1 b, d). We used these EFDs of forewings and hindwings as two different datasets during statistical analyses. To obtain size components we estimated the centroid sizes from the scaled images implementing '*Momocs*' (Bonhomme et al. 2014).

Landmark based analysis for wing venation: We used the same 156 specimen images to quantify venation patterns. Landmarks were placed at vein intersections around the cell of both fore- and hind- wings. We used 8 landmarks for both forewing and hindwing cells. All landmarks were digitized using *TpsDig2* (Rohlf and Slice 2010). For both wings, landmark configurations were superimposed using a generalized Procrustes super-imposition (Rohlf and Slice 1990). The resulting coordinates in the tangent space were used as shape data. All morphometric and statistical analyses were conducted using R package '*geomorph*' (Adams and Otarola-Castillo 2013) and '*Momocs*' (Bonhomme et al. 2014). The two wings were analyzed separately in subsequent analyses.

Statistical analyses: Firstly, we performed principal component analysis (PCA) on both EFDs of outlines (Fig X1) and Procrustes coordinates of vein junctions (Fig X2) for both forewing and hindwing. Further statistical analyses based on PCs were done only including those taxa which have at least 5 specimens each. To understand the extent of difference between mean wing shapes and venation across taxa we used the PCs cumulatively explaining 90% of the total variance to calculate the Euclidean distances in the morphospace between each butterfly

taxon mean shape (Table SX1, SX2), using the R package '*geomorph*' (Adams and Otarola-Castillo 2013). Additionally, a nonparametric multivariate analysis of variance (MANOVA) associated randomized residual permutation test is done to estimate the significance of the distances (Collyer et al. 2015). Moreover, to understand the extent of overlaps in wing shape outlines and venation among taxa in morphospace we used linear discriminant analysis (LDA) based predictions on the principal components (Fig SX2, SX3) using R package '*Momocs*' (Bonhomme et al. 2014). To understand the covariation between forewings and hindwings on both outline EFDs and venation Procrustes coordinates datasets, we calculated RV scores which is a multivariate analogue of Pearson's coefficient (Escoufier 1973; Klingenberg 2009) using package '*FactoMineR*' (Lê S. et al. 2008). We also performed two-block partial least squares (PLS) regression when the RV coefficient was significantly different from zero. To account for a potential allometric effect, PLS between forewing and hindwing was performed on the residuals of a multivariate regression of shape variables on centroid size, including all individuals considering EFDs and Procrustes distance based datasets separately (Fig SX4a, SX5a). Additionally, to find the covariation between outline shape and venation configuration we calculated RV and performed two-block PLS between outline EFDs and venation-based Procrustes co-ordinates taking forewings and hindwings separately (Fig X3). To understand relation between forewing and hindwing size we performed correlation test between centroid sizes of forewing and hindwing using R base package '*stats*'(Fig SX4b, SX5b). We also obtained comparative visualization by thin plate spline-based transformation procedure in '*Momocs*' for pairwise comparisons of wing shape outlines (Fig SX6)and spatial segregation in landmark positions for venation patterns (Fig SX7).

Wing colour pattern analyses: Forewing and hindwing upper side images resized to (300px × 300px) from the same 156 specimens were used for colour pattern analysis. We analyzed forewings and hindwings separately. In most of the participant taxa for both fore- and hind-wing upper side colour patterns are mostly represented with dark background and interspersed light color patches. Therefore, we have chosen position of dark colour in the forewing and hindwing to extract wing colour patterns. To align images, we used an automated intensity-based registration method on two-dimensional raster objects associated with a colour thresholding procedure which requires RGB value and cutoff value inputs for each taxon to separate darker background from lighter colour patches. Wing colour patterns were extracted, aligned, and analyzed using the R package 'patternize' (Van Belleghem et al. 2018). Background noise in the wings that were co-extracted with the colour patterns were masked. Next, the registration procedure calculates transformations from all rasters to a target raster based on either intensity patterns. Raster stacks of specimens were taken for further analysis. Within taxon variation in the pattern were obtained and visualized as heatmaps by summing the separate binary raster stacks of forewing and hindwing (Fig SX8) using *sumRaster* function (Van Belleghem et al. 2018). Subsequently proportion of the background pattern compared to the total wing is calculated, using the *patArea* function for both forewing and hindwing of each taxa (Van Belleghem et al. 2018) (Fig SX8). Finally, principal component analysis (PCA) was performed on the transformed binary dataset obtained from raster of each sample (Fig X4). The PCA also provides visualization of the predicted colour pattern changes along the PC axis. To understand the extent of difference between mean colour patterns we used the PCs to calculate the Euclidean distances and estimated significance using randomized residual permutation procedure as stated in the geometric morphometry section (Collyer et al. 2015) (Table SX3). For the Euclidean distance,

relative area calculations, and heatmap visualization, we considered only those taxa which have at least 5 specimens each.

Results

Wing outlines: Principal component 1 based on the forewing EFDs exhibits shape changes of broad to narrow elongated wings with produced apical region explaining 52.7% of the total shape variation. PC2 of the forewing delineate the range from rounded tornal region with curved dorsum to more angular tornal region with flatter dorsum explaining 17.7 % of the total shape variation (Fig X1a). PC1 of hindwings majorly describes the variation in the curve of termen region, explaining 39.9% of the total shape variation, whereas PC2 describes the range from narrow to broader wings, explaining 29.4% of the total shape variation (Fig X1b). Pairwise MANOVA result on the PCs exhibit show mostly significant differences in both forewing and hindwing mean shapes across taxa (Table SX1). The LDA predictions show major overlap [original taxa : predicted taxa] among *avalokita* : *brahma*, *gautama* : *brahma*, *naima* : *davidis*, and *avalokita* : *brahma* in forewing (Fig SX2). In the hindwing, LDA prediction show major overlaps among *avalokita* : *lhatso*, *gautama* : *brahma*, *lactoris* : *davidis*, and *sudassana* : *brahma* (Fig SX2). Transformation maps of forewings show major intra taxon variation within *brahma*, *funebris*, and *lhatso*. Hindwing does not exhibit similar extent of intra taxon variation as forewing. Pairwise comparison among forewing mean shapes show most shifts near termen and tornus region. In hindwing pairwise comparison changes in dorsum, tornus and costa region (Fig SX6).

Venation configurations: PCA on the forewing Procrustes coordinates exhibit 52.2% variation explained in the first two PC axes where shape changes along PC1 and PC2 show spatial compression and dilation in vein junctions with landmark 2, 3, 4, 5 (Fig X2a). In hindwing, first two PC axes explain 55.7% of the total shape variation and shape changes along first two PCs show shifts near vein junctions with landmark 4, 5, 6, 7, 8 (Fig X2b). Pairwise MANOVA results show in forewing *lhastso* and *naima* are very distinct in mean venation pattern, while in hindwing gautama, naima and sudassana are the most distinct ones in mean shape. The LDA predictions show major overlap [original taxa : predicted taxa] among *avalokita* : *buddha*, *buphonas* : *davidis*, *gautama* : *buddha*, *lactoris* : *davidis* and *sudassana* : *brahma* in forewing (Fig SX3). In hindwing, the major overlaps are among *buddha* : *brahma*, *buphonas* : *davidis*, *gautama* : *davidis*, and *lactoris* : *davidis* (Fig SX3). Transformation map reflect the similar spatial shifts in landmark position as visualized in shape changes along PC axes (Fig SX7).

Covariation between forewing and hindwing, and between outline and venation: Covariation between forewing and hindwing outline shapes taking effects of size in consideration was low across all individuals (RV coefficient = 0.212, P<0.0001). First PLS axes explain 77.66% of the total covariation show clearly less correlation according to taxa (Fig SX4a). Correlation between forewing and hindwing centroid size is also low (R^2 = 0.05, P = .007) (Fig SX4b). Some taxa (*brahma*, *buddha*, *gautama* and *sudassana*) is with more variation in hindwing centroid size than forewing, whereas other taxa show more allometric relation between forewing and hindwing centroid size (Fig SX4b). Contrarily, landmark configurations describing venation patterns in forewing and hindwing show low covariation (RV coefficient = 0.186, P<0.0001), while the first PLS axes describing 49.63% of the total covariation show very high correlation (Fig SX5a). Similarly, centroid size of the cell region of the forewing and hindwing are also highly correlated (R^2 = 0.45, P<0.0001) (Fig SX5b). Covariation between outline EFDs and Procrustes co-ordinates delineating vein junctions is low across all individuals in both forewing (RV coefficient = 0.127, P<0.0001) and hindwing (RV

coefficient=0.08, *P* = 0.0002). First PLS axes explaining 69.66% and 69.12% of total covariation in forewing and hindwing respectively show low taxa specific correlations(Fig X3a, b).

Colour patterns:

We extracted the dark background surrounding the paler patches delineating wing patterns. In the PCA on forewing colour patterns, the first two axes exhibit changes in position of lighter patches (Fig X4a). In hindwing the first PC axes show presence or absence of a particular colour pattern phenotype rather than change in spatial distribution of interspersed lighter patches, which is shown along PC2 (Fig X4b). In the morphospace, we see a more constrained forewing colour pattern compared to variable and overlapping hindwing colour pattern across taxa. Pairwise MANOVA show significant differences in mean colour pattern between almost all taxa pair for both forewing and hindwing (Table SX3). Intra-taxon variation is shown in the heatmap visualization along with relative area of the dark background on the wing of each taxon (Fig SX8). These results show in forewing *davidis* and *lactoris* have relatively less dark background area along with larger light patches which is distinct than other taxa, while in hindwings *lactoris* and *lhatso* have the similar patterns.

References

Adams, D. C., and E. Otarola-Castillo. 2013. Geomorph: an R package for the collection and analysis of geometric morphometric shape data. *Methods in Ecology and Evolution.* 4:393–399.

Bonhomme, V., S. Picq, C. Gaucherel, and J. Claude. 2014. Momocs: outline analysis using R. *Journal of Statistical Software*. 56:1–24.

Collyer,M. L., D. J. Sekora, and D. C. Adams. 2015. A method for analysis of phenotypic change for phenotypes described by high-dimensional data. *Heredity.* 115:357–365.

Escoufier, Y. 1973. Treatment of vector variables. *Biometrics*. 29:751–760.

Ferson, S., F. J. Rohlf, and R. K. Koehn. 1985. Measuring shape variation of two-dimensional outlines. *Systematic Zool*ogy 34:59–68.

Klingenberg, C. P. 2009. Morphometric integration and modularity in con-figurations of landmarks: tools for evaluating a priori hypotheses. *Evolution & Development*. 11:405–421.

Kuhl, F. P., and C. R. Giardina. 1982. Elliptic Fourier features of a closed contour. *Computer Graphics and Image Processing*. 18:236–258.

Lê S, J. Josse, F. Husson. 2008. "FactoMineR: A Package for Multivariate Analysis." *Journal of Statistical Software*. 25(1), 1–18.

Rohlf, F. J., and D. Slice. 1990. Extensions of the Procrustes method for the optimal superimposition of landmarks. *Systematic Zool*ogy 39:40–59.

Rohlf, F. J., and D. Slice. 2010. TpsDig 2.16. Department of Ecology and Evolution, State University of New York at Stony Brook, Stony Brook, NY.

Van Belleghem S.M., R. Papa, H. Ortiz-zuazaga, F. Hendrickx, C.D. Jiggins, W.O. Mcmillan, and B.A. Counterman. 2018. patternize: an R package for quantifying colour pattern variation. *Methods in Ecology and Evolution.* 9:390-398.

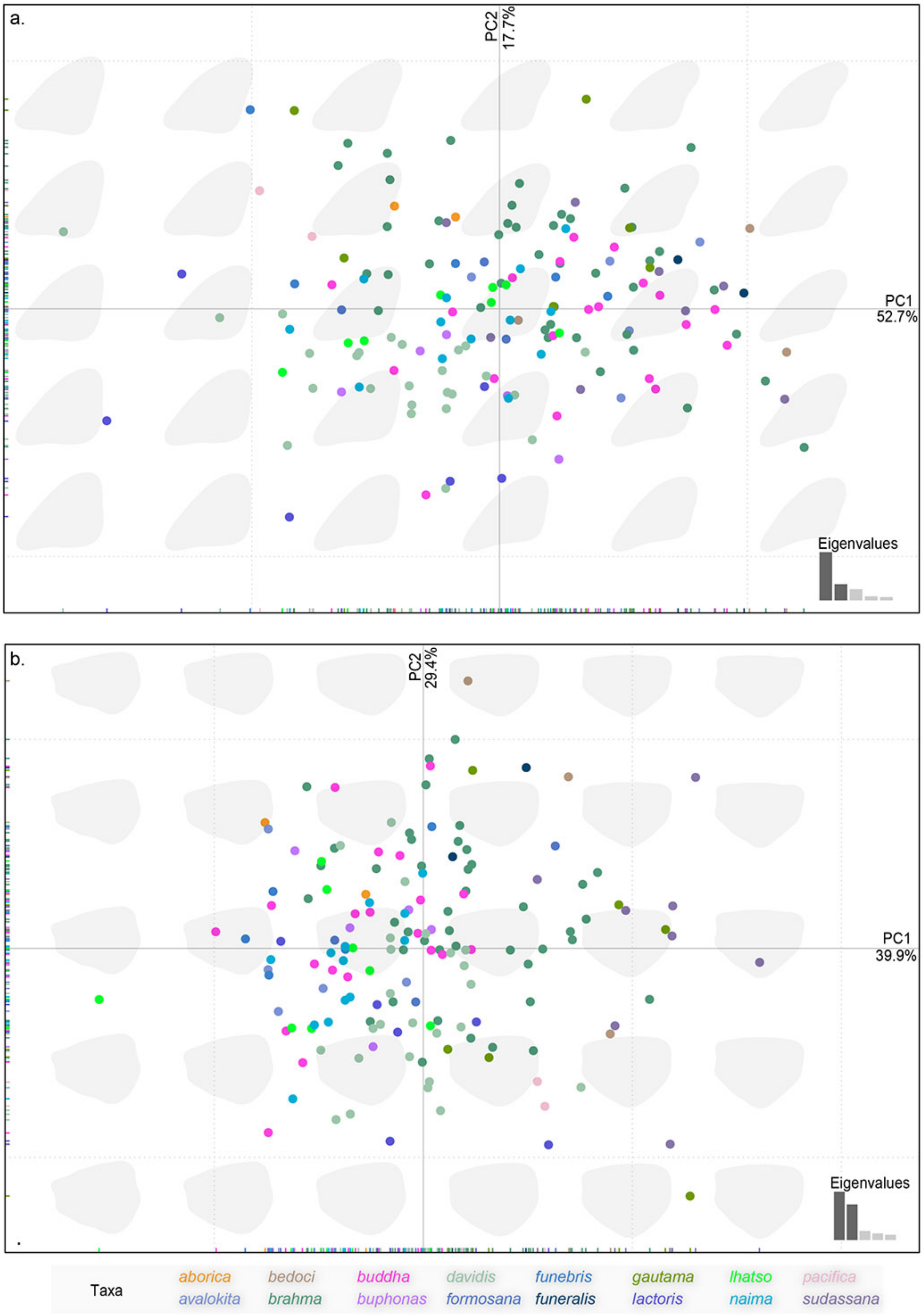

Fig X1: Forewing (a) and hindwing (b) morphospace plot (based on PCA on elliptical Fourier descriptors extracted from wing outlines); all the individuals are colour coded according to taxa. Predicted wing shapes show changes in outline shapes along principal components 1 and 2. In each plot Eigenvalues are representing relative variance explained by first five principal components (PCs). For the first two PCs the bars are dark and percentage of variation explained are indicated at termini of both axes.

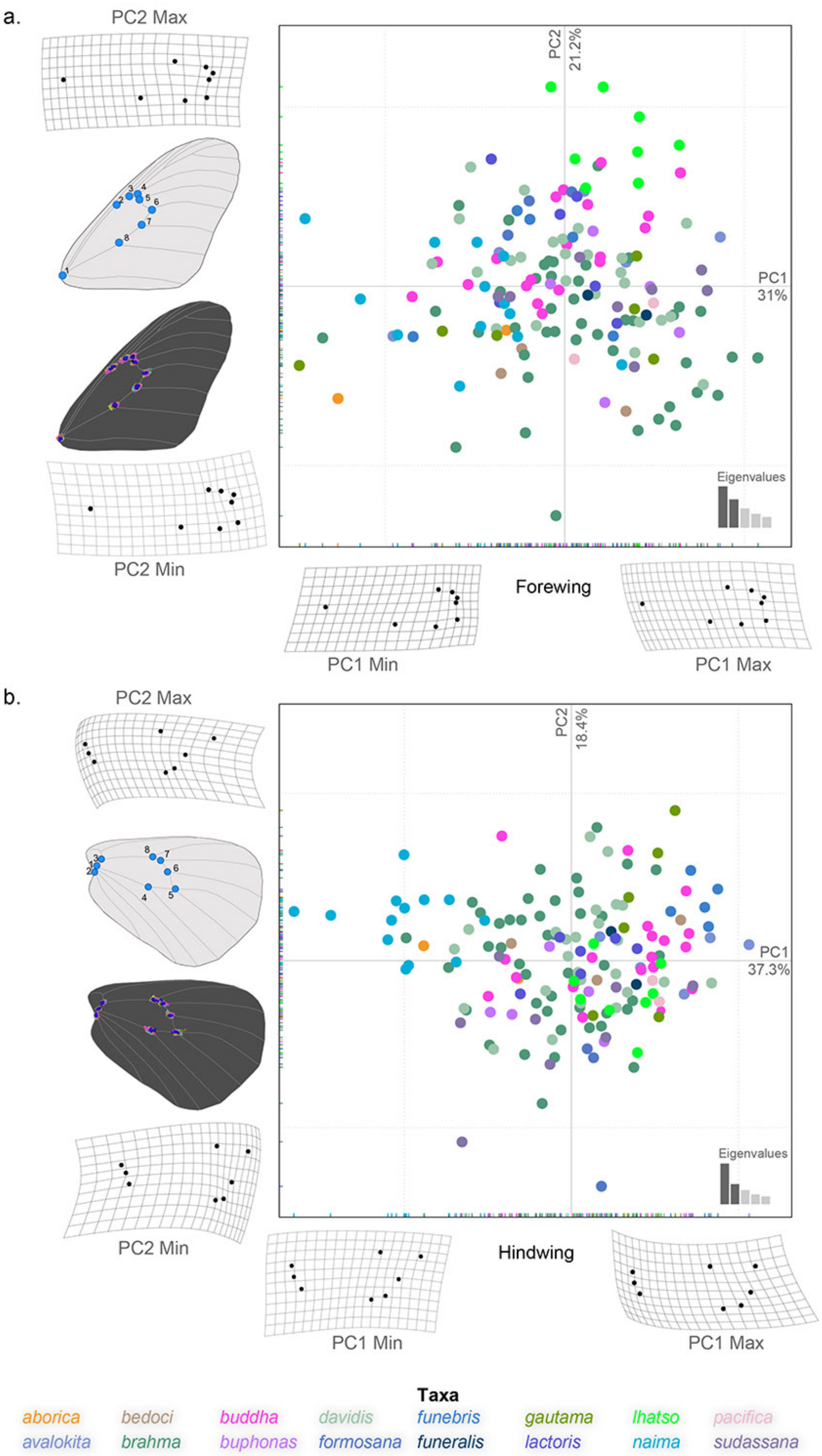

Fig X2: Forewing (a) and hindwing (b) morphospace plot (based on PCA on Procrustes coordinates of landmarks on vein junction around cell region); all the individuals are colour coded according to taxa. Predicted shapes with transformation grid show changes in vein junctions along principal components 1 and 2. In each plot Eigenvalues are representing relative variance explained by first five principal components (PCs). For the first two PCs the bars are dark and percentage of variation explained are indicated at termini of both axes.

Cartoon of mean landmark configurations (light grey) and assemblage of landmarks from all the specimens (dark grey) for both forewing and hindwing are also shown.

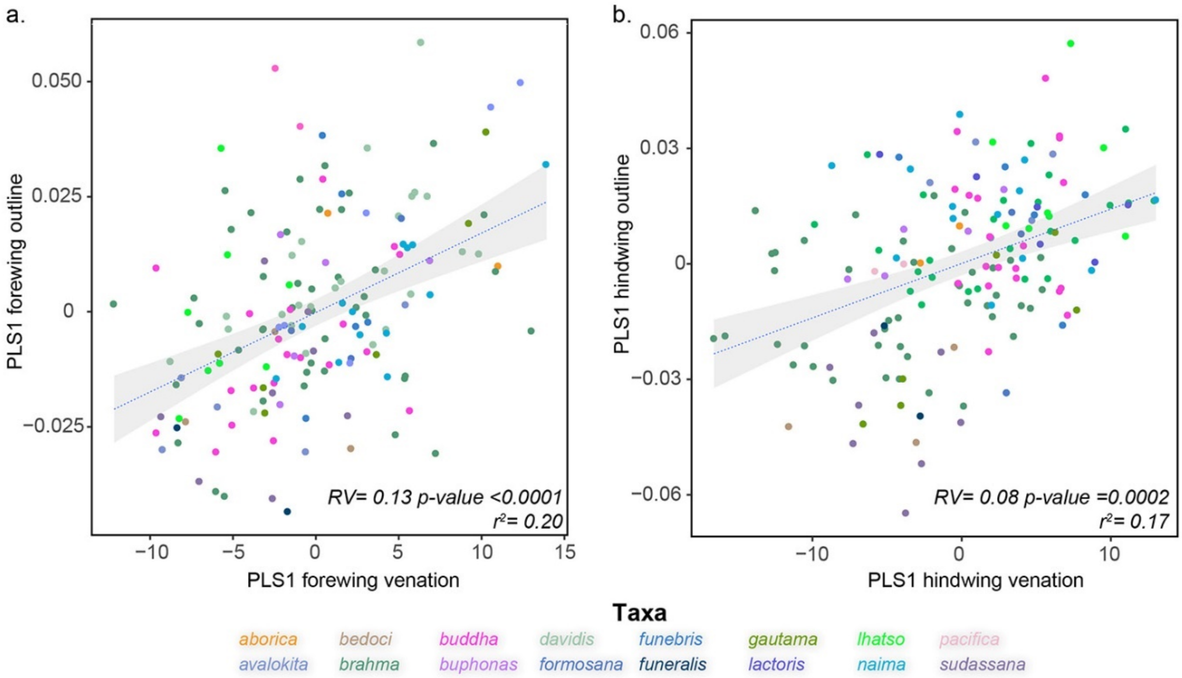

Fig X3: Partial least square regression results showing covariation between outline shape predictors and wing venation landmarks around cell region in both forewing (a) and hindwing (b) for all individuals. All individuals are colour coded according to taxa. The PLS1 axes explain 69.66% and 69.12% of total variation in forewing (a) and hindwing (b).

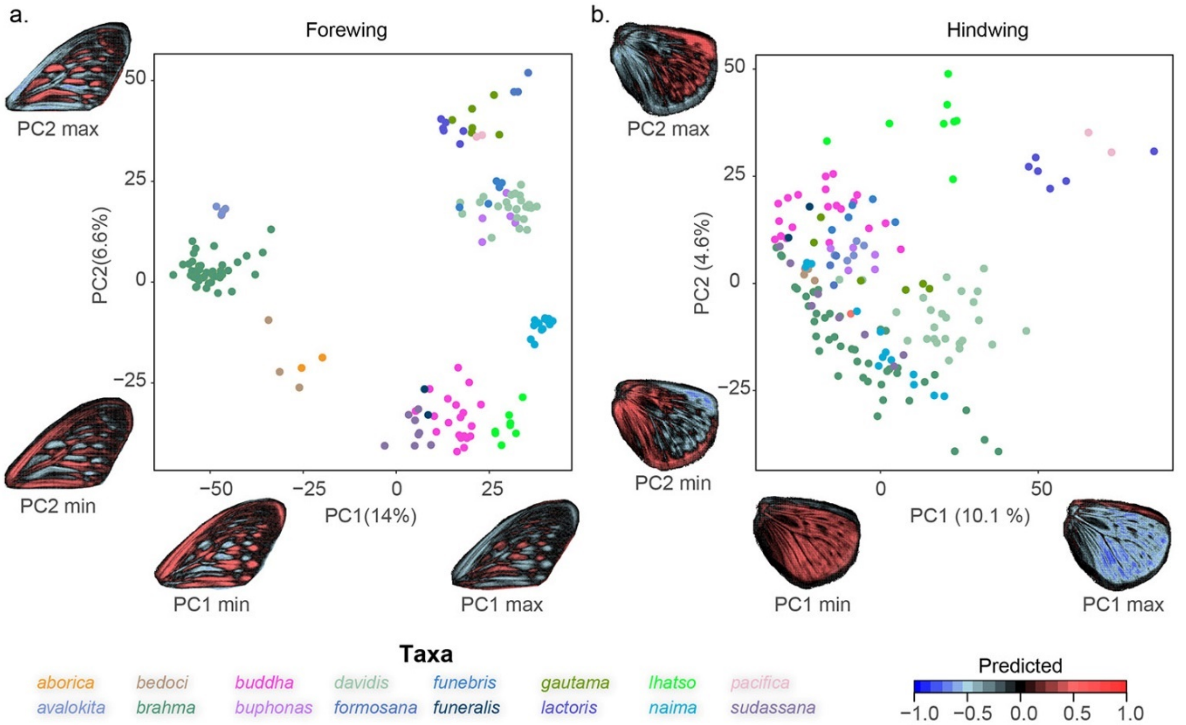

Fig X4: PCA on raster stacks of dark background colour pattern in forewing (a) and hindwing (b). Predicted colour pattern changes along first two PC axes are also shown. In the cartoon, positive values presenting a higher predicted presence of the specific pattern are in red and negative values presenting the absence of the pattern are in blues.

Supplementary figures and tables

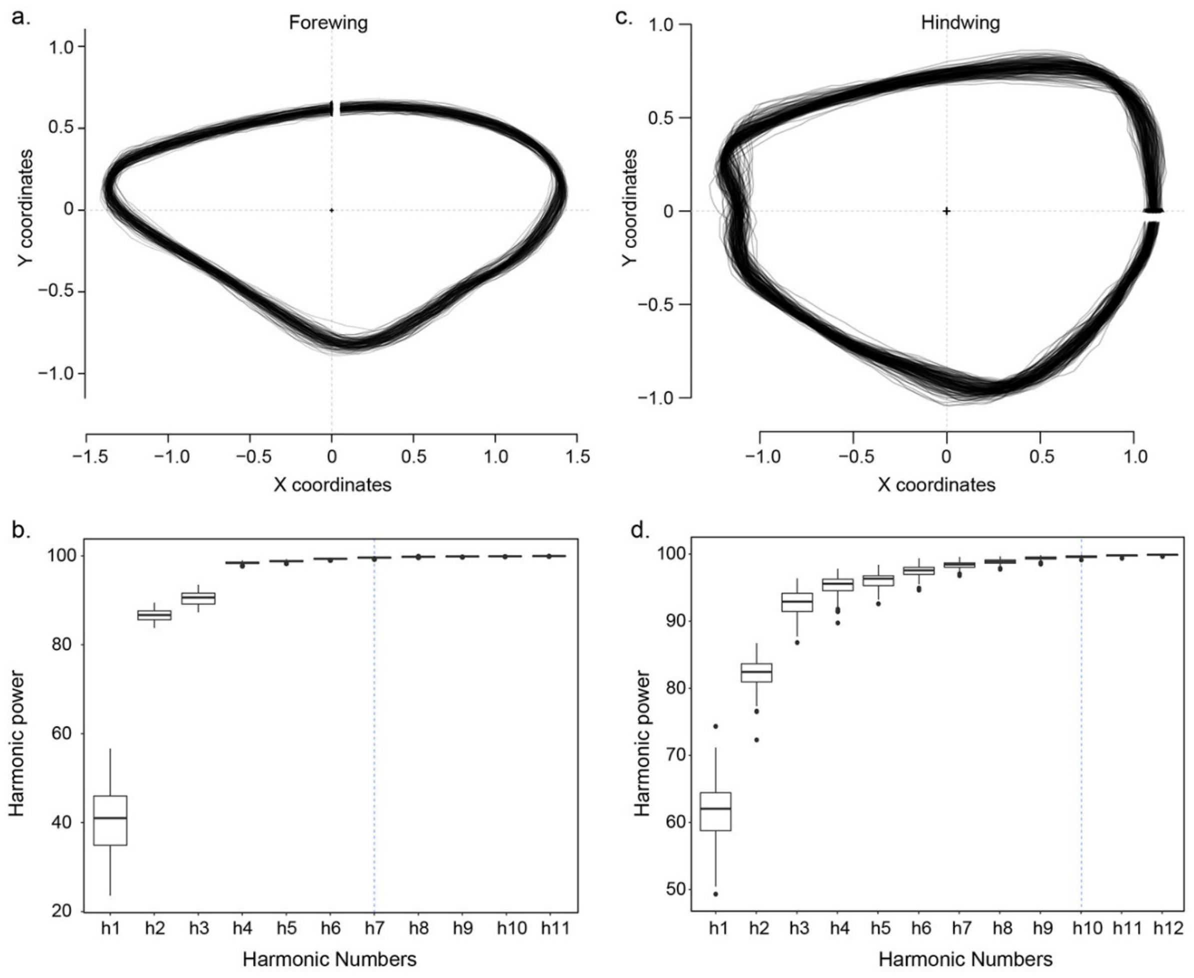

Fig SX1: Outline shape stacking of forewing (a) and hindwing (c). Boxplots showing cumulative harmonic power (harmonics required to accurately delineate a shape) for both forewing (b) and hindwing (d). Blue dotted line along the harmonics indicate the total number of harmonics chosen to define wing outline with harmonic power >0.90.

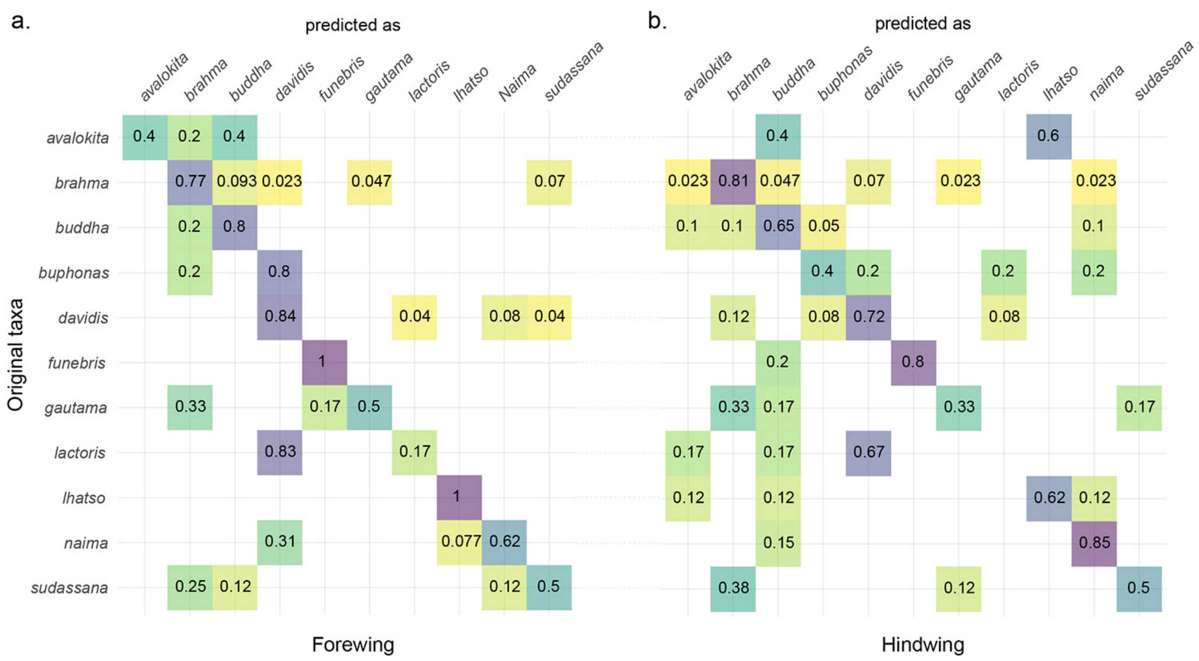

Fig SX2: LDA prediction across taxa based on PC axes cumulatively explaining more than 90% of total variation. In the matrix, the frequency of forewing (a) and hindwing (b) predictions are shown to exhibit taxa with overlapping wing outlines. Frequencies are colour coded as yellow-green-violet representing lower to higher frequencies.

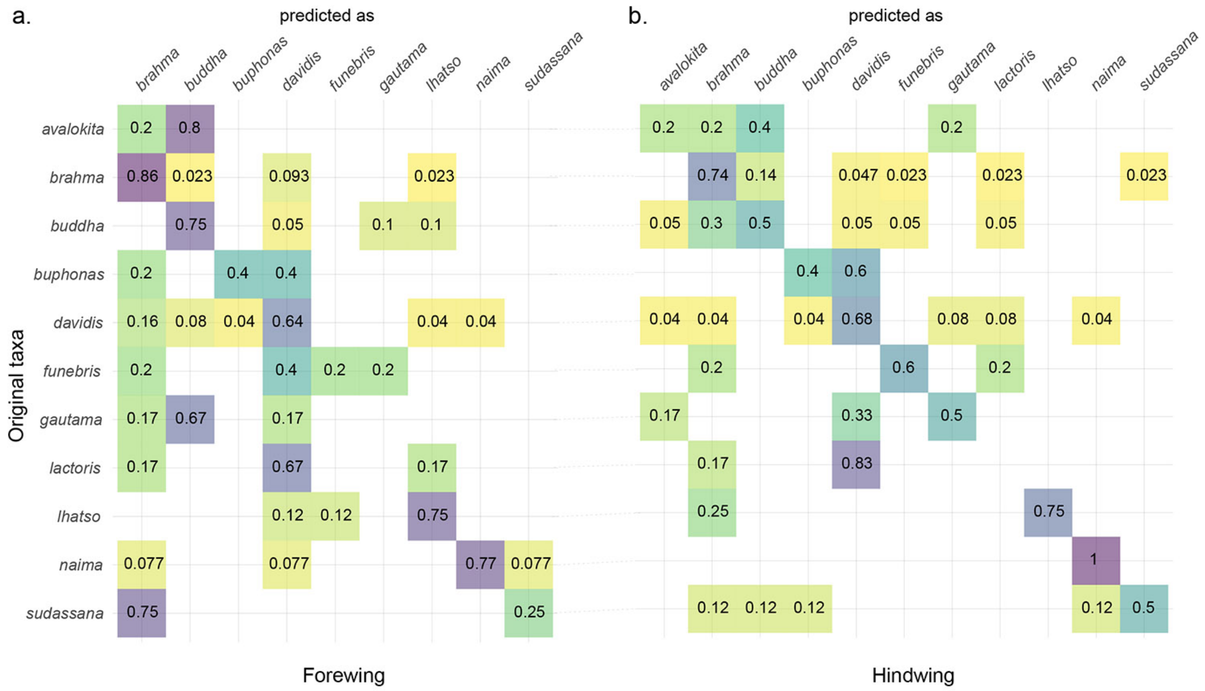

Fig SX3: LDA prediction across taxa based on PC axes cumulatively explaining more than 90% of total variation. In the matrix, the frequency of forewing (a) and hindwing (b) predictions are shown to exhibit taxa with overlapping vein junctions in the cell region. Frequencies are colour coded as yellow-green-violet representing lower to higher frequencies.

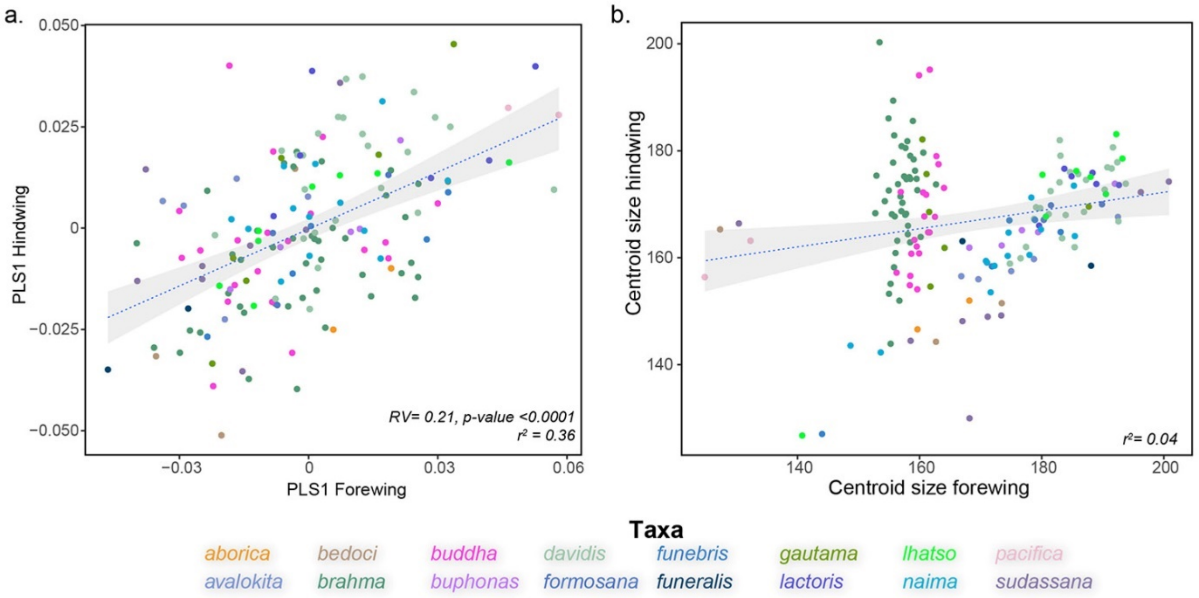

Fig SX4: Partial least square on EFDs of wing outlines showing covariation between forewing and hindwing across individuals (a). The correlation between forewing and hindwing centroid size (b).

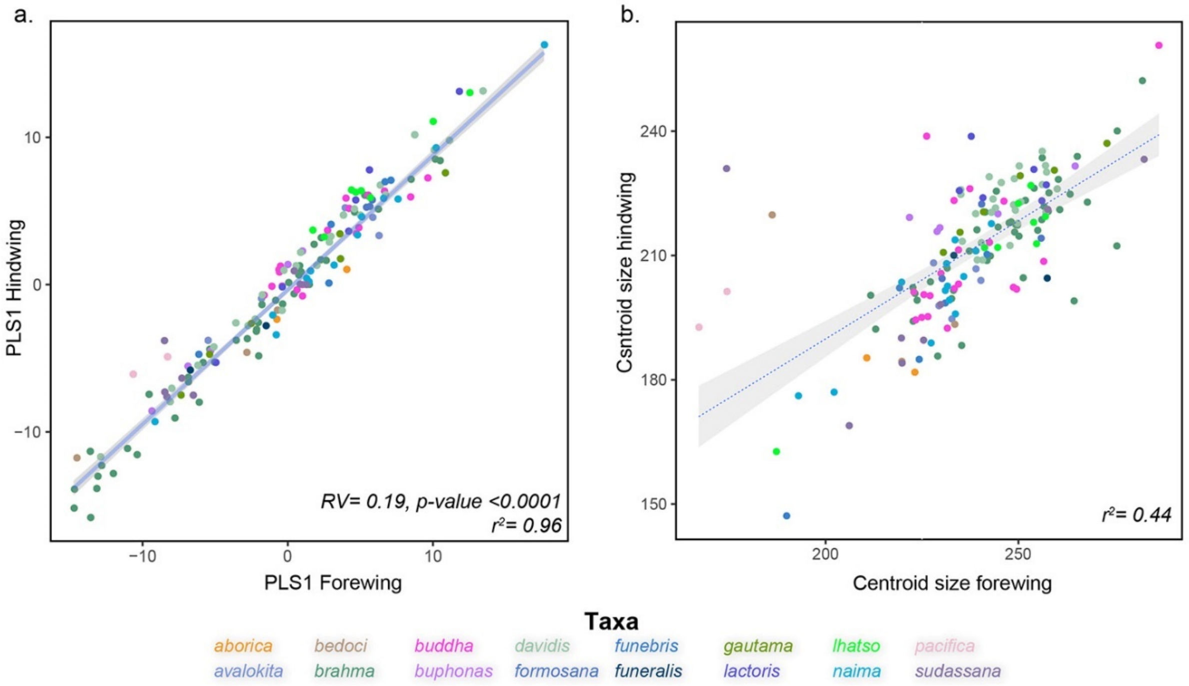

Fig SX5: Partial least square on EFDs of transformed coordinates of vein junction around cell region showing covariation between forewing and hindwing across individuals (a). The correlation between centroid size of cell region in forewing and hindwing (b).

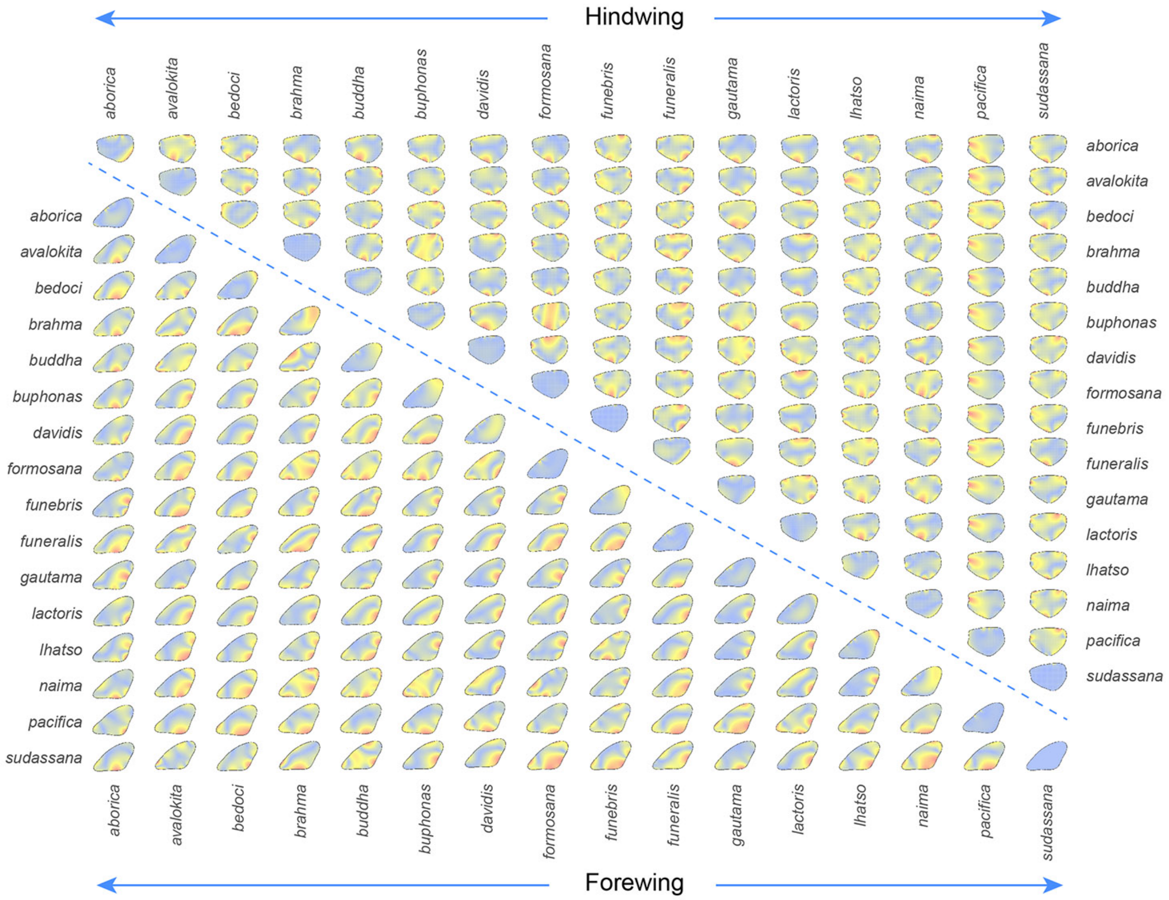

Fig SX6: Pairwise mean shape comparison among all 16 taxa. In the matrix, lower and upper triangular halves represent the forewing and hindwing differences, respectively. Each half showing mean wing outline shape differences in a transformation map where in a scale of blue-yellow-red, area of wings with least or no changes are mapped blue and most variable regions are mapped red.

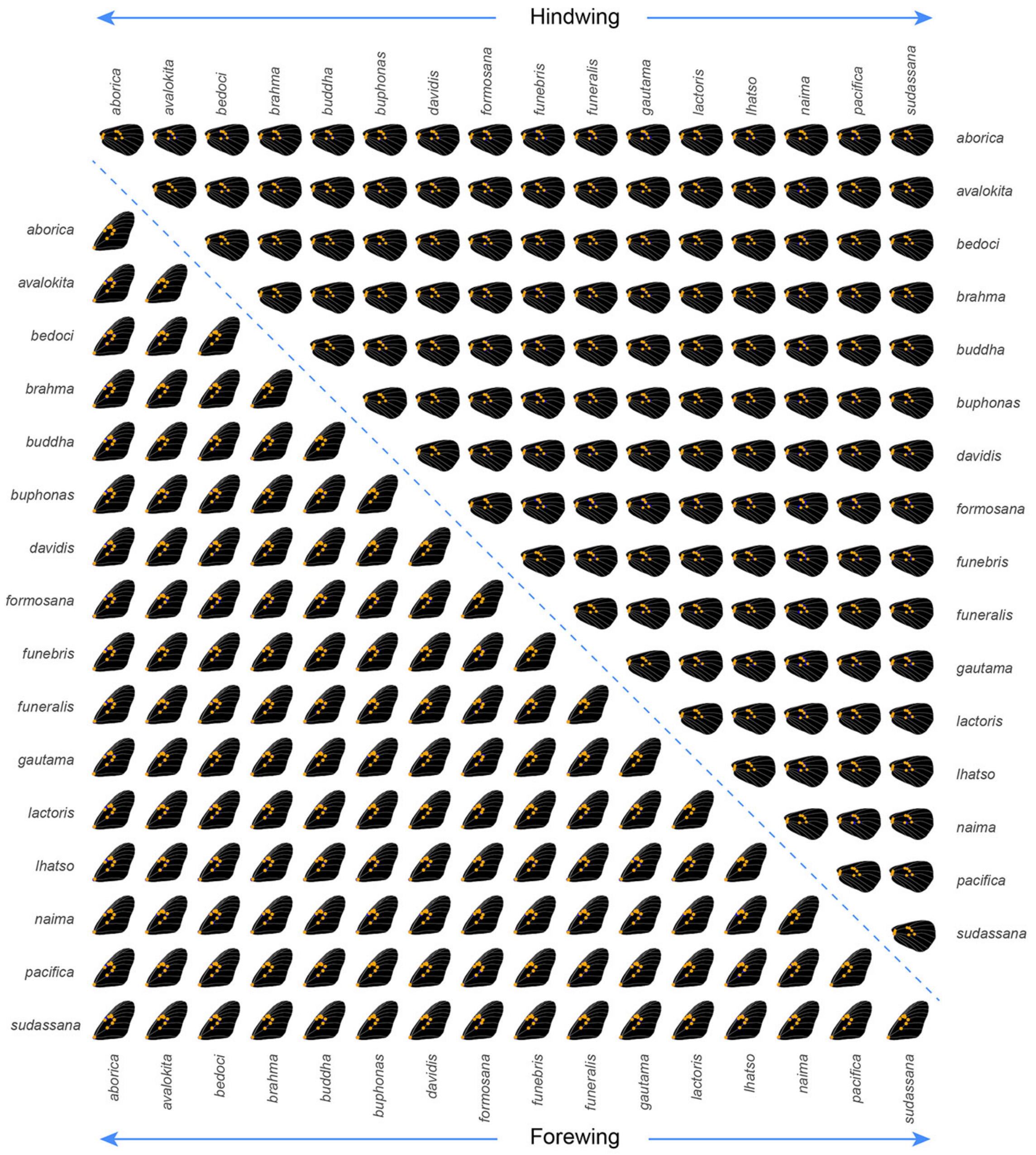

Fig SX7: Pairwise mean shape comparison among all 16 taxa based on mean landmark coordinates of vein junctions. In the matrix, lower and upper triangular halves represent the forewing and hindwing differences, respectively. Each half showing spatial differences of vein junctions around cell between each taxa pair, where one set of landmarks are orange (reference taxon) and the other set blue (comparing taxon).

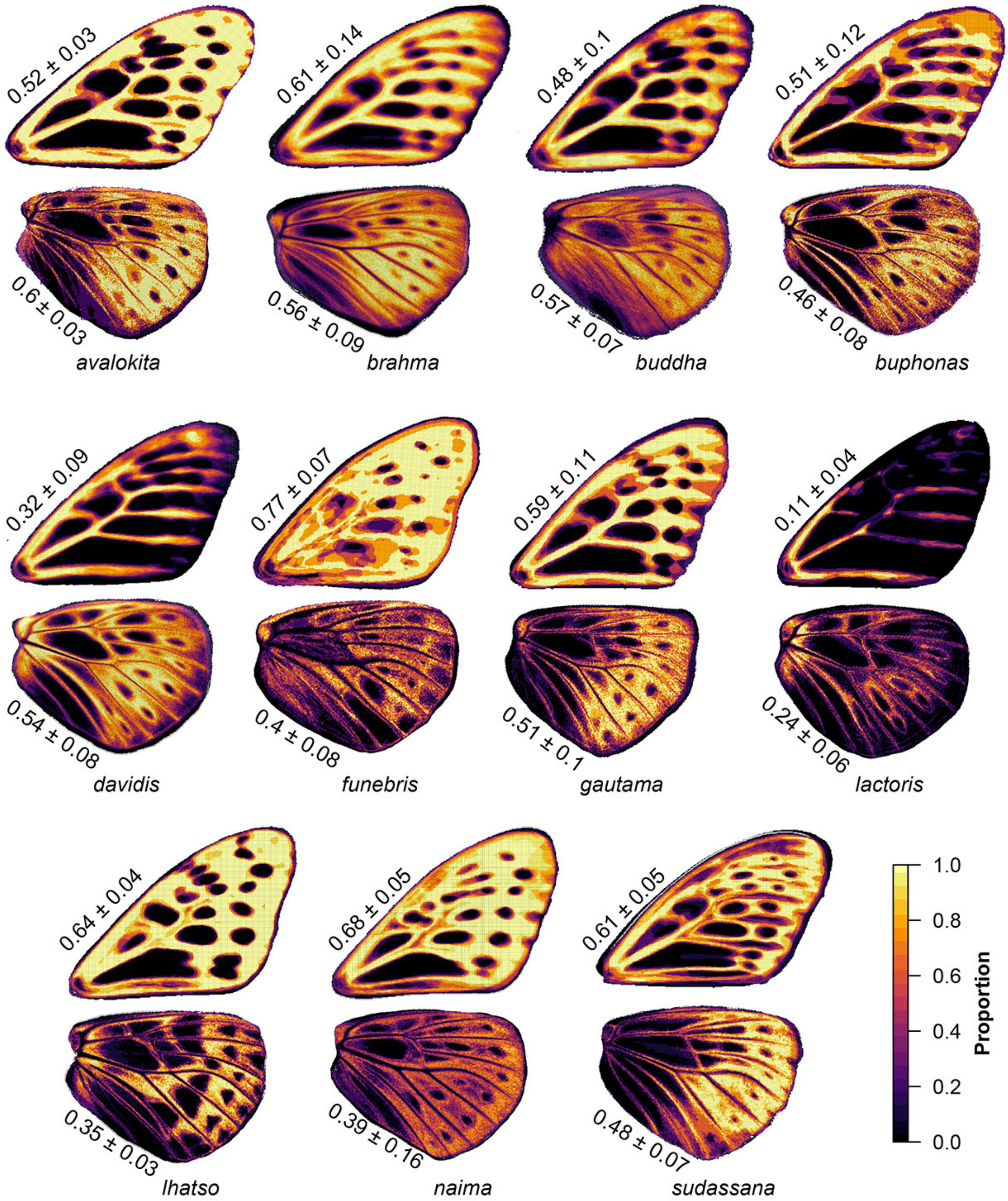

Fig SX8: Quantification of dark background colour pattern in 11 taxa. Heatmaps demonstrate the consistency of colour pattern yellow indicating consistent presence of patterns and red-dark purple gradient indicating less consistent presence. The black areas represent the lighter interspersed patches. Mean and standard deviation of relative size (proportion of the wing) of background colour pattern are also shown.

**Taxa**	*avalokita*	*brahma*	*buddha*	*buphonas*	*davidis*	*funebris*	*gautama*	*lactoris*	*lhatso*	*naima*	*sudassana*
avalokita	NULL	**0.034**	0.014	0.024	**0.028**	0.020	**0.056**	**0.033**	0.011	0.018	**0.073**
brahma	0.019	NULL	**0.022**	0.024	**0.025**	**0.033**	**0.029**	**0.032**	**0.035**	**0.028**	**0.044**
buddha	0.017	**0.015**	NULL	0.017	**0.023**	0.020	**0.046**	**0.029**	0.017	0.016	**0.062**
buphonas	**0.030**	**0.025**	0.023	NULL	0.019	0.024	**0.046**	0.027	0.028	0.019	**0.061**
davidis	**0.037**	**0.030**	**0.030**	0.011	NULL	**0.035**	**0.038**	0.013	**0.031**	**0.022**	**0.055**
funebris	**0.036**	**0.029**	**0.032**	0.031	**0.028**	NULL	**0.055**	**0.039**	0.019	0.020	**0.072**
gautama	**0.028**	**0.020**	**0.025**	**0.032**	**0.033**	0.022	NULL	**0.039**	**0.055**	**0.046**	0.027
lactoris	**0.049**	**0.042**	**0.041**	0.021	0.014	**0.039**	**0.046**	NULL	**0.033**	**0.027**	**0.057**
lhatso	**0.035**	**0.033**	**0.030**	0.026	**0.024**	0.015	**0.027**	**0.035**	NULL	0.018	**0.072**
naima	**0.028**	**0.024**	**0.023**	0.016	**0.018**	0.023	**0.023**	**0.030**	0.018	NULL	**0.066**
sudassana	0.014	0.016	0.015	**0.030**	**0.037**	**0.036**	**0.026**	**0.049**	**0.037**	**0.028**	NULL

Hindwing

Forewing

Table SX1: Euclidean distances between the mean wing outline shape of 11 taxa. In the matrix lower and upper half represents forewing and hindwing, respectively. Significant distances are marked in bold. The significance of the distances was based on a MANOVA associated randomized residual permutation procedure.

**Taxa**	*avalokita*	*brahma*	*buddha*	*buphonas*	*davidis*	*funebris*	*gautama*	*lactoris*	*lhatso*	*naima*	*sudassana*
avalokita	NULL	**0.049**	0.028	**0.057**	**0.046**	0.039	0.038	0.044	0.035	**0.106**	**0.059**
brahma	**0.039**	NULL	**0.026**	0.035	**0.034**	**0.067**	**0.052**	0.031	**0.038**	**0.063**	**0.036**
buddha	0.013	**0.040**	NULL	**0.045**	**0.037**	**0.052**	**0.036**	0.033	**0.037**	**0.087**	**0.046**
buphonas	**0.052**	0.032	**0.049**	NULL	0.025	**0.076**	**0.057**	0.025	0.042	**0.068**	0.033
davidis	0.032	**0.034**	**0.026**	0.029	NULL	**0.058**	**0.039**	0.012	**0.038**	**0.073**	**0.048**
funebris	0.031	**0.038**	0.029	0.043	0.025	NULL	**0.053**	**0.056**	**0.052**	**0.113**	**0.083**
gautama	0.032	0.032	0.033	0.047	0.031	0.030	NULL	0.040	**0.056**	**0.101**	**0.069**
lactoris	0.043	**0.042**	0.036	0.032	0.018	0.028	0.043	NULL	0.037	**0.073**	**0.042**
lhatso	**0.053**	**0.069**	**0.050**	**0.064**	**0.056**	**0.059**	**0.076**	**0.052**	NULL	**0.087**	**0.042**
naima	**0.057**	**0.050**	**0.054**	**0.059**	**0.050**	**0.045**	**0.044**	**0.049**	**0.079**	NULL	**0.073**
sudassana	**0.045**	0.027	**0.042**	0.028	**0.035**	**0.046**	**0.046**	0.041	**0.059**	**0.054**	NULL

Hindwing

Forewing

Table SX2: Euclidean distances between the vein junction positions on the cell region of 11 taxa. In the matrix lower and upper half represents forewing and hindwing, respectively. Significant distances are marked in bold. The significance of the distances was based on a MANOVA associated randomized residual permutation procedure.

Taxa	*avalokita*	*brahma*	*buddha*	*buphonas*	*davidis*	*funebris*	*gautama*	*lactoris*	*lhatso*	*naima*	*sudassana*
avalokita	NULL	81.525	86.733	99.091	94.763	111.219	86.240	113.150	98.501	92.355	87.526
brahma	85.558	NULL	64.725	77.103	61.300	92.872	80.545	106.226	95.075	72.114	78.883
buddha	115.346	84.973	NULL	85.681	76.187	91.727	92.976	106.201	94.129	80.859	89.265
buphonas	122.710	100.768	81.020	NULL	80.058	94.150	90.962	101.605	100.331	83.425	88.868
davidis	113.781	89.430	73.619	80.679	NULL	95.876	86.690	97.909	94.490	80.101	84.543
funebris	128.535	102.280	91.991	85.471	81.598	NULL	103.507	89.627	96.915	83.135	106.121
gautama	125.601	99.866	97.184	86.180	84.854	85.922	NULL	101.339	90.401	79.540	72.114
lactoris	123.038	105.865	101.945	96.954	91.262	113.671	99.487	NULL	87.601	83.083	104.727
lhatso	131.819	105.732	83.651	103.605	86.054	108.345	115.366	118.125	NULL	81.182	90.462
naima	129.669	104.473	86.471	93.068	80.198	98.325	94.382	114.914	86.674	NULL	78.463
sudassana	116.359	83.671	55.992	94.095	83.561	101.048	104.256	106.253	85.976	93.818	NULL

Hindwing

Forewing

Table SX3: Euclidean distances between wing colour patterns of 11 taxa. In the matrix lower and upper half represents forewing and hindwing, respectively. Significant distances are marked in bold. The significance of the distances was based on a MANOVA associated randomized residual permutation procedure.
