# Supplementary material for: DNA barcodes from over-a-century-old type specimens shed light on the taxonomy of a group of rare butterflies (Lepidoptera: Nymphalidae: Calinaginae)
Source: PLoS One. 2024 Jul 17;19(7):e0305825. doi: 10.1371/journal.pone.0305825 (PMC11253935; doi:10.1371/journal.pone.0305825)
Supplement: S3 File — (PDF) [file pone.0305825.s004.pdf]

SUPPLEMENTARY INFORMATION S4

Morphotaxonomy of subfamily Calinaginae, with special reference to *Calinaga aborica*

RICHARD I. VANE-WRIGHT

Scientific Associate, Insects Division, Department of Science, Natural History Museum, London SW7 5BD, UK

During 1969–1971, I commenced a revision of *Calinaga* – for three reasons. At that time, the limits of the Nymphalidae were uncertain, with the danaines, morphines, acraeines, libytheines and other 'brush foots' often regarded as separate families. As the curious *Calinaga* had, for example, even been included in the Papilionidae (e.g. Kirby, 1871: 510 – see Watson, 1899), and was regarded by de Nicéville (1900) as an aberrant danaine, it represented a key taxon for any reassessment. Second, was subfamilial status within a broad concept of the Nymphalidae, as proposed by Ehrlich (1958), warranted? Third, did the group consist of "a single monobasic genus", as Ehrlich (1958: 352) also suggested? In pursuit of other goals, I subsequently abandoned this project – even though I had by then carried out extensive research. At the time I concluded that acceptance of the family Nymphalidae *sensu lato*, to include the Calinaginae, Danainae, Morphinae, Acraeinae and Libytheinae, *inter alia*, as subfamilies, was the best arrangement – and that *Calinaga* was a polytypic genus, comprising, as I later reported, "about 8 species" (Vane-Wright, 2003: table 22.1, 487–88).

The opportunity is taken here to present some of my earlier morphotaxonomic findings in light of the latest molecular results, as reported in the main paper.

Calinaginae

Calinaginae Moore, 1895: 220. Type genus, by monotypy: *Calinaga* Moore, [1858].

Membership of the Nymphalidae. Tricarinate antennae, which bound two continuous sulci or paired depressions on each segment (Jordan, 1898; Ackery *et al*., 1999); prothoracic legs greatly reduced in both sexes, not used for walking (this latter condition is not unique to the family, but characteristic nonetheless).

Membership of the satyroid subclade. Mesothoracic anepisternum present as a distinct sclerite (Ehrlich, 1958). Ehrlich (1959 – phenetics), Freitas & Brown (2004 – parsimony), and Kawahara *et al*. (2023 – maximum likelihood) all placed, in effect, the Calinaginae as sister to Satyrinae *sensu lato* (thus including Morphini, Brassolini etc. + Charaxinae), at the base of the satyroid subclade.

Diagnostic characters of the Calinaginae. The following combination of characters is unique within the Nymphalidae: male genitalia with a superuncus but without a gnathos; female protarsus with small but well-formed claws; vein 3A of forewing free at base (Ehrlich, 1958); antennae short (about 30% of forewing length) and stout, with the last ten or so flagellar segments thickened to form a long club, and two oblate sensory depressions on every flagellar segment, one within each ventromesial sulcus (Ackery *et al.*, 1999). In addition, the eyes are densely hairy, and the strongly arched HW vein M_3 is notable (de Nicéville, 1886: 142).

Among the butterflies, a superuncus (which arises from the 8th tergite, not the tegumen: Klots, 1970: 118) is perhaps best known in the Papilionidae. In the Nymphalidae it is very unusual – although a supposedly comparable condition occurs in the Libytheinae (Ackery *et al.*, 1999). Given the many uncertainties affecting our understanding of the homologies of Lepidoptera genitalia, further research would be welcome. The complex antennal surface (Vane-Wright, 2015: 68, and unpublished) also offers potential for the discovery of uniquely diagnostic characters for the genus – and even specific differences (e.g. the apparently sensory depressions of *Calinaga aborica* are far smaller than those of *C. davidis* – RIVW, pers. obs.).

Calinaga Moore

Calinaga Moore, [1858]: 162. Type species, by monotypy: *Calinaga buddha* Moore, [1858]. Cowan: 1975 [date of publication].
Calinago Herrich-Schäffer 1864: 112, 132. *Lapsus calami.*
Callinaga Jordan, 1898: pl. 15 (legend). *Lapsus calami.*

Diagnostic characters of Calinaga. The adults of all *Calinaga* are all very similar anatomically, such that division into two or more genera would seem totally unjustifiable. Thus the diagnostic characters of the genus are the same as those of the subfamily. An additional character is the conspicuous red, orange or golden 'hair' that clothes both the thoracic dorsum and pleural sclerites of all know species, at least in part.

At species level, the variation in the extent of the coloured thoracic hairs is of some taxonomic value (see key to species groups). The male genitalia are rather uniform (Lang, 2012), but would repay more extensive and systematic comparison – and the same is, I believe, true for the female genitalia, including variation in the well-developed signae of the bursa copulatrix (Lang, *op. cit*.; Vane-Wright, unpublished). The antennae, as noted, may also differ, at least quantitatively, between species or species-groups.

Calinaga aborica Tytler, 1915

(Figs S4.1–S4.7)

Calinaga aborica Tytler, 1915: 511, pl. 3, fig. 21. LECTOTYPE male, Abor Hills, India (NHMUK), here designated [examined].
Calinaga aborica; Lang, 2012: 50, pl. 3 (fig. 30), pl. III (fig. 13); Sondhi *et al.* (2016).
Calinaga (*davidis*) *aborica*; Tshikolovets, 2020: 76–78, pl. 26 (10 figs).

Calinaga aborica was described from two male specimens (syntypes), collected "by Captain Porter" on the Dihang River [a section of the Brahmaputra], Abor Hills, in June and July [1913] (Tytler, 1915: 511). Originally in Sir Harry Tytler's own collection, soon after his death in May 1939 these specimens were obtained by the nymphalid specialist Arthur Hall (1873–1952) – one male subsequently being passed by Hall to the NHMUK, London, the other to the Booth Museum, Brighton, UK (as part of the Hall Collection). The male in the NHMUK is herewith designated Lectotype, and has the following labels: /♂ Abor Hills 6.13 [June 1913]/Tytler Coll. 1940/Type/A. Hall. B.M. 1942-11./B.M. Type No. Rh. 17201/Lecto-type/Calinaga aborica Tytler Lectotype ♂ det. R.I. Vane-Wright 1971/BMNH(E)#665228/. This designation is consistent with previous type restrictions (e.g. Sondhi *et al.*, 2016). The male in the Booth Museum, dated [July 1913], has been labelled as a Paralectotype.

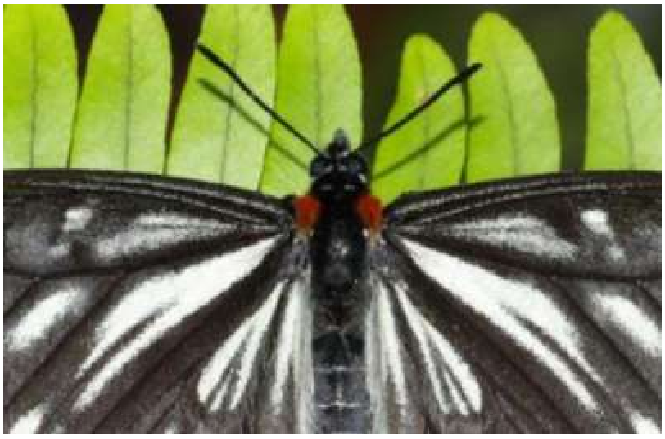

Figure S4.1. *Calinaga aborica.* Uniquely in this species, the red or orange hairs and scales visible on the thoracic dorsum are entirely restricted to the tegulae (see text). In all other *Calinaga* species the red or orange hairs occur more widely on the dorsum. ©The Trustees of the Natural History Museum, London (Creative Commons License 4.0 https://creativecommons.org/licenses/by/4.0/).

Diagnosis: A relatively small species of *Calinaga* that is separable from all other members of the genus by at least four characters:

- Red hairs of thoracic dorsum restricted (uniquely) to the tegulae (Fig. S4.1; ["sides at body at junction of the forewings red" – Tytler, 1915: 510])
- Hindwing discocellular veins m_1–m_2 and m_2–m_3 form a slight but distinct obtuse angle of about 150–160° (Fig. S4.2; ["lower discocellular of cell of hindwing distinctly concave and not straight, ending at the junction of vein 3 and 4 and not above it" – Tytler, *loc. cit.*]; other species have the end-points of these two veins more or less in a straight line – 180°)
- Centres of postdiscal and submarginal pale spots in hindwing cell CuA_2 further apart than the corresponding spots in cell CuA_1, and the spots in CuA_1 further apart than the corresponding spots in cell M_3 (Fig. S4.3ai, bi) – a pattern not found in any other member of the genus)
- Oblate sensory depressions of the antennal sulci are small, occupying approximately the basal half of each flagellar segment.

Female unknown.

Calinaga aborica aborica Tytler, 1915
(Fig. S4.3a, S4.4)

Barcode Index Number: BOLD:ACZ3798

Diagnosis. The nominate subspecies is relatively melanic compared to many *Calinaga* taxa (one of its vernacular names is the Dark Freak). Despite this, there is considerable variation in the size and extent of the separate pale pattern elements, with the result that individuals vary in overall 'brightness'. Two colour pattern features appear to be diagnostic for separation from the only other currently recognised subspecies:

- On the underside, although the dark-greyish ground colour is relieved by scattered yellow scales, these are sparse, notably at the base of hindwing cells CuA_1 and CuA_2; in contrast, in subsp. *naima* the yellow scales on the hindwing underside are far more numerous, or dense, notably at the base of cells CuA_1 and CuA_2.
- Subtriangular, submarginal pale spot in cell M_1 on the hindwing upperside is significantly wider than the postdiscal streak in the same cell, to the extent that if virtual anterior and posterior tangential lines are drawn to touch these two marks, the lines converge before the base of the wing (Fig. S4.3a; also

Fig. S4.4e). In subsp. *naima* these virtual lines, even if convergent, do not actually converge until extended beyond the base of the hindwing.

Typically but variably (Fig S4.4), the pale postdiscal streaks in forewing upperside cells R_5, M_1 and M_2 are slightly less well-developed (the length of the streak in cell R_5 *ca* 2–4 mm), the pale streaks in hindwing upperside cell R_1 are generally less prominent, the distal areas of hindwing upperside cells 2A and 3A are often strongly infuscated at the margin; and on the hindwing underside the distal triangular spot in cell R_1 is usually small (sometimes wholly obsolete), often highly dyslegnic, although it can be up to *ca* 3.5 mm in length.

Forewing length (male): mean 43.47 mm [n = 4; observed range 42.6–44.2 mm, SD 0.797]; Lang (2012), however, reported 41–42 mm for material from Mêdog, Tibet. Female unknown.

Material directly examined: lectotype (NHM, London), paralecotype (Booth Museum, Brighton), one male from Kabu, Mêdog County, Tibet, collected by F.M. Bailey (American Museum Natural History, New York).

Distribution: **India, Arunachal Pradesh**: Abor Hills, Dihang River [Brahmaputra], June and July 1913 (type material [*ca* 28.4°N, 94.7°E); Pakke Tiger Reserve, East Kameng District, 21.v.2015 (illustrated by Anonymous, 2024 [*ca* 27.1°N, 92.8°E]); Siang, Siang District, 22.v.2022 (illustrated by Anonymous, 2024 [*ca* 28.4°N, 94.7°E]); near Yinkiong, Upper Siang District, 22.v.2022, 12.v, 18.v., 19.v.2023 (illustrated by Anonymous, 2024 [*ca* 28.6°N, 95.0°E]); Gobuk, Upper Siang District, 18.v.2023 (illustrated by Anonymous, 2024 [28.569°N, 95.102°E]).

China, Tibet, Nyingchi, Mêdog County: 'Kapu', Yarlung Tsangpo river [Upper Brahmaputra], 6 June 1913, 3000 ft., F.M. Bailey (1 male, AMNH, New York, examined; see Bailey, 1914 [= Kabu Village, Mêdog County, 29.473°N, 95.453°E]); Mêdog, 710–1450 m, v.1983 (9 males, IZCAS, Beijing; see Lang, 2012: 50, one illustrated [29.325°N, 95.333°E]); 'Xigonghu' (Tshikolovets, 2020: pl. 26, fig.3/4 [Mêdog area]).

Myanmar, N Sagaing District: Tarung Hka River, 10.v.2008, local collector [working on behalf of the late Prasobsuk Sukkit – Adam Cotton, pers. comm. 7 Feb. 2024] (2 males, Tor Vergata University, Rome; photograph examined [*ca* 27.2°N, 96.8°E, at 1,050 m – for a map showing the Tarung river, Sagaing District, see Lambert, 1937: 311]).

Flight period and altitudinal range: records are for May–July; there are scant data on altitude, with only two records, both at about 1000 m. Probably mid-montane.

Calinaga aborica naima **Vane-Wright**
(Fig. S4.3b, S4.5)

urn:lsid:zoobank.org:act:773B49ED-C54E-4D76-8E61-267F5722320C
Barcode Index Number: BOLD:ACZ3798

Diagnosis. Subspecies *naima* differs from the western, nominotypical populations as indicated above, and as in the diagnostic description formally presented in the main part of this paper (Todisco *et al.* 2024) .

Forewing length (male): mean 40.96 mm [n = 17; observed range 37.6–43.1 mm, SD 1.441]; Female unknown.

A note on Kingdon Ward's localities (Fig. S4.6). The holotype and one of the paratypes were collected by Frank Kingdon Ward in the Seinghku Valley, 'Upper Burma', on 27th and 17th May 1926, at 6500 ft and 5000 ft, respectively. If you plot the coordinates given on the data labels of the holotype (28.5 N 97.35 E.), interpreted either as DD or DMS, you come to points in Nyingchi Province, Tibet, a few kilometers north-west of the Myanmar/China border. By comparing Kingdon Ward's (1930) own account of the area, including his map (Fig. S4.6), with information in the ornithological gazetteer of Thwin *et al.* (2011), it becomes clear that these two localities are close to both Tibet and India, but are in Myanmar. The full name of the river is Seinghku Wang – which appears on the 1954 US Army Map Service sheet NH 47-13 (downloadable at

https://commons.wikimedia.org/wiki/File:Txu-oclc-10552568-nh47-13.jpg). Based on this map, the point at which the Seinghku Wang reaches 6500 ft (≈ 1980 m), the type locality, lies at *ca* 28.07 N 97.48 E (DD) – approximately 10 km west of Tazungdam, 10 km east of the border with India, and less than 30 km from the easternmost part of the Mishmi Hills in Arunachal Pradesh. In contrast, the DMS coordinates given on some of the paratypes collected in the Mishmi Hills during the 1928 Percy Sladen Expedition, of which Kingdon Ward was a participant (Kingdon Ward, 1930), appear to be correct.

Distribution: **India, Arunachal Pradesh**: Mishmi Hills, 3 March 1928, 2000 ft. [F. Kingdon Ward] (2 males, NHMUK, *ca* 28.0 N, 96.0 E); Mishmi Hills, Delei Valley, 11 May 1928, 4500 ft. [F. Kingdon Ward] (1 male, NHMUK [*ca* 28.35 N, 96.62 E]); Mishmi Hills [above Delei Valley], 13 June 1928, 10,000 ft. [F. Kingdon Ward] (2 males, NHMUK [*ca* 28.35 N, 96.62 E]).

Myanmar, N Kachin State: Seinghku Wang Valley, 27 May 1926, 6500 ft., F. Kingdon Ward (holotype male, NHMUK [*ca* 28.07 N 97.48 E]); Seinghku Wang River, 17 May 1926, 5000 ft., F. Kingdon Ward (1 male, NHMUK [*ca* 28.0 N 97.50 E]); Adung Valley [upper Tamai River], 14 May 1931, 6000 ft., Lord Cranbrook (3 males, NHMUK [*ca* 28.14 N, 97.7 E]); Mairudam, NE Putao, 5–14 May 1998, Prasobsuk Sukkit (4 males, 3 currently in Adam Cotton collection, the fourth in Howard Grisham collection [not located; very approximately 27.50 N, 98.25 E; "1800 m"]). Also seen: photograph of 1 male from "Chudu Razi Hills, 30 miles E Kawnlangphu" [very approximately 27.10 N, 98.6 E], and 1 male from "Wanzewong-Ngawar". Kachin State, figured by Shizuya *et al.* (2011: 26).

DISCUSSION

Is the division of the very distinctive *Calinaga aborica* Tytler into two subspecies justifiable? Tshikolovets (2020), without explicitly saying so, evidently thought not. He treated the undoubted "variability of *C. aborica*" (*op. cit.*, p. 78, fig. O – see Fig. S4.7) as individual variation, not geographic. However, his figure O has several locality labelling errors which, once corrected, does reveal the geographical variation described here (Fig. S4.7). But even if this is accepted, it must then be questioned if this variation is clinal or discontinuous. There is some suggestion that it represents no more than a west (dark) to east (bright) stepped cline, the most westerly known *C. aborica aborica* being somewhat darker than those from the Abor Hills region, and Kachin *aborica naima* perhaps being a little brighter than those from Mishmi Hills. In addition, one known Tibetan *C. aborica aborica*, identified as such by the two diagnostic characters, is otherwise bright, but seems likely to be an exception – e.g. compare this specimen, Fig. S4.4e,f, with Tshikolovets, 2020, pl. 26, figs 3–6). However, the evidence is also consistent with a significant west–east discontinuity between the Abor and Mishmi hills populations – which are separated by no more than about 80 km – and in Myanmar to the south, in a similarly narrow west–east separation, between the Sagaing area (*aborica aborica*) and the northern Kachin populations (*aborica naima*).

The Mishmi Hills are located in the region where the Indian and Eurasian plates meet (Salvi *et al.*, 2020), in a complex section of the Eastern Himalayan Syntaxis (EHS) known as the Tuting-Tidding Suture Zone. To the south lies the Myanmar plate, also part of the EHS, separated from the Indian plate by the major Sagaing Fault (Salvi *et al.*, 2020; Morishita *et al.*, 2023). Thus the division of *Calinaga aborica* into two sets of populations, nominotypical *aborica* to the west of this plate boundary region, subspecies *naima* to the east, may have some earth-historical basis.

Subspecific taxonomy is often vexed (see Vane-Wright, 2020, for a review with respect to butterflies). One of the problems rarely addressed by butterfly taxonomists – who sometimes

seem obsessed by the practice of naming subspecies – is that this is generally done on the basis of one character system only – typically, minor variations in wing coloration, pattern and size. In his landmark book, Hennig (1966: 54) stated that "There are ... practically no rules for differentiating subspecies." Taken at face value, this seems an odd complaint from someone so committed to natural-order systematics, whereby taxa are not defined by some edict or established by rote, but are simply and uniquely discovered. However, his objection is made clear by means of an important theoretical diagram (*op. cit.* fig. 13, p. 57). If differences observed among morphological, physiological, ethological and other character systems are combined into a single 'holomorphological' index, no congruent pattern corresponding to biological reality necessarily emerges. In the present case, we can at least claim that the separation of *Calinaga aborica* into east/west subtaxa, on the admittedly limited current evidence, reflects a congruent pattern derived from both wing pattern and molecular data.

In conclusion, with respect to justification, is the division proposed of any utility? We answer in the affirmative, insofar as it appears to raise interesting questions potentially open to test – notably, has the evolution of *Calinaga aborica*, since its origin as a separate clade, been affected by its occurrence across a major tectonic boundary region – and, if so, when and how? In this context, Adam Cotton (pers. comm, 7.ii.2024) interestingly points out that there are subspecies of Papilionidae that occur in both the Mêdog area of S Tibet and in northern Sagaing – but this is by no means the case for all swallowtail subspecies. Could there be several biogeographic patterns in this area that, perhaps, reflect different time periods in the relatively recent geological history of this extraordinary region?

ACKNOWLEDGEMENTS

Repeated from main paper: RIVW is greatly indebted to past curators and former colleagues at a number of European and North American museums, with holdings of *Calinaga* butterflies that he was able to access in the 1970s. With respect to revived work on the genus over the past few months, RIVW wishes to thank Robyn Crowther, Jon Earle, Niall Briggs and Charlotte Birkett at the Natural History Museum, London, for their assistance. Special thanks are due to Adam Cotton, Thailand, for his knowledge, help and insight regarding these insects.

FIGURES S4.2 – S4.7 follow:

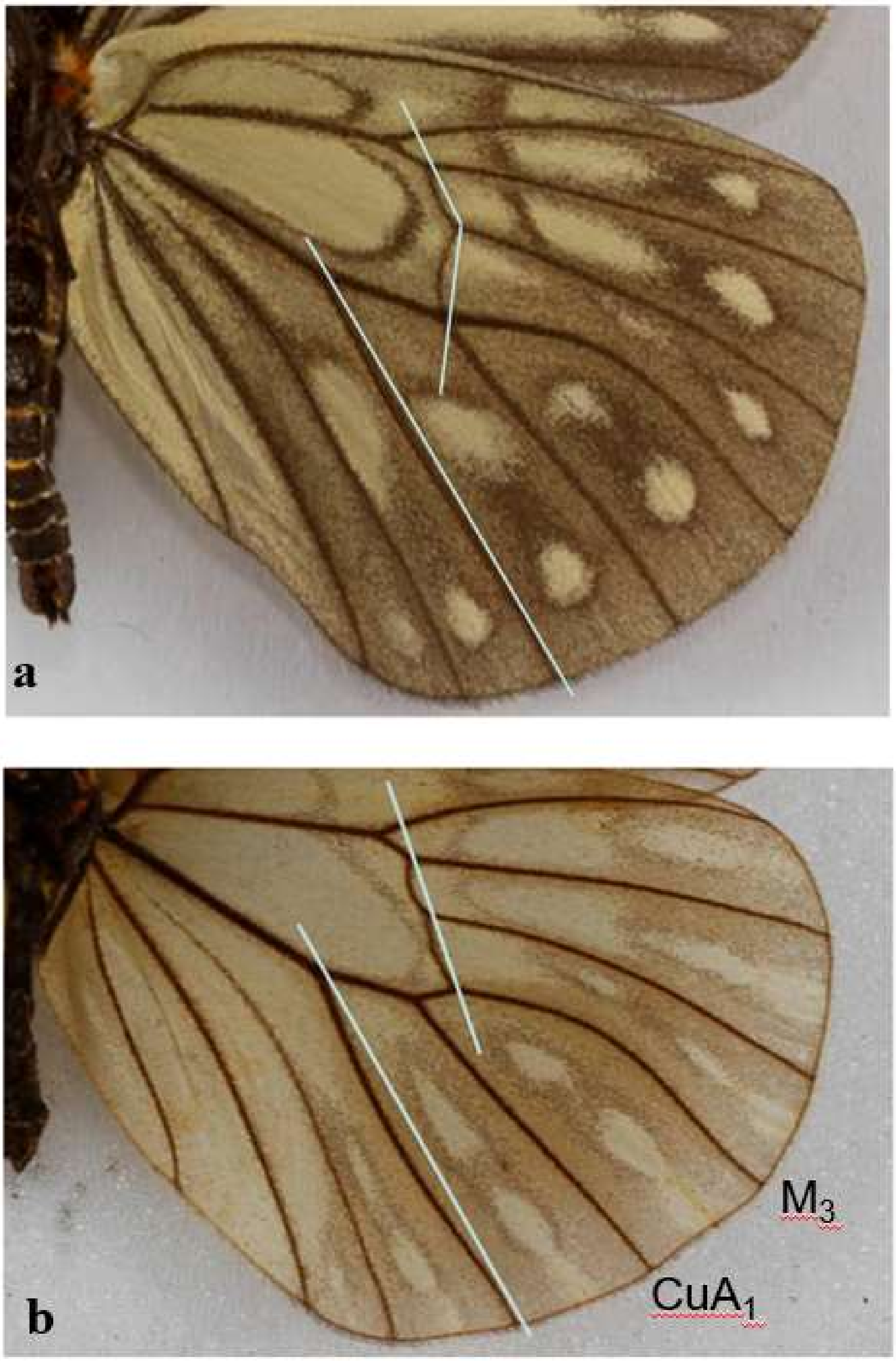

Figure S4.2. Hindwing underside of *Calinaga*. **a**, *C. aborica*: Uniquely in this species, the discocellular veins m_1–m_2 and m_2–m_3 form a slight but distinct obtuse angle of about 150–160° (anterior angled blue indicator lines); **b**, *C. davidis*: in all other *Calinaga* species, the branching points of veins m_1–m_2 and m_2–m_3 lie in or very close to a straight line (see text). In addition, note that in the great majority of *C. davidis* sensu lato vein CuA$_2$ is distinctly bowed posteriorly (posterior blue indicator line), whereas in *C. aborica* and all other *Calinaga* species it is only slightly so, straight, or even slightly bowed anteriorly. Note also that in the great majority of *C. davidis*, m_2–m_3 joins vein M$_3$ distintly beyond its branching point with CuA$_1$, unlike *C. aborica* and the majority of *Calinaga*

individuals belonging to other species. ©The Trustees of the Natural History Museum, London (Creative Commons License 4.0 https://creativecommons.org/licenses/by/4.0/).

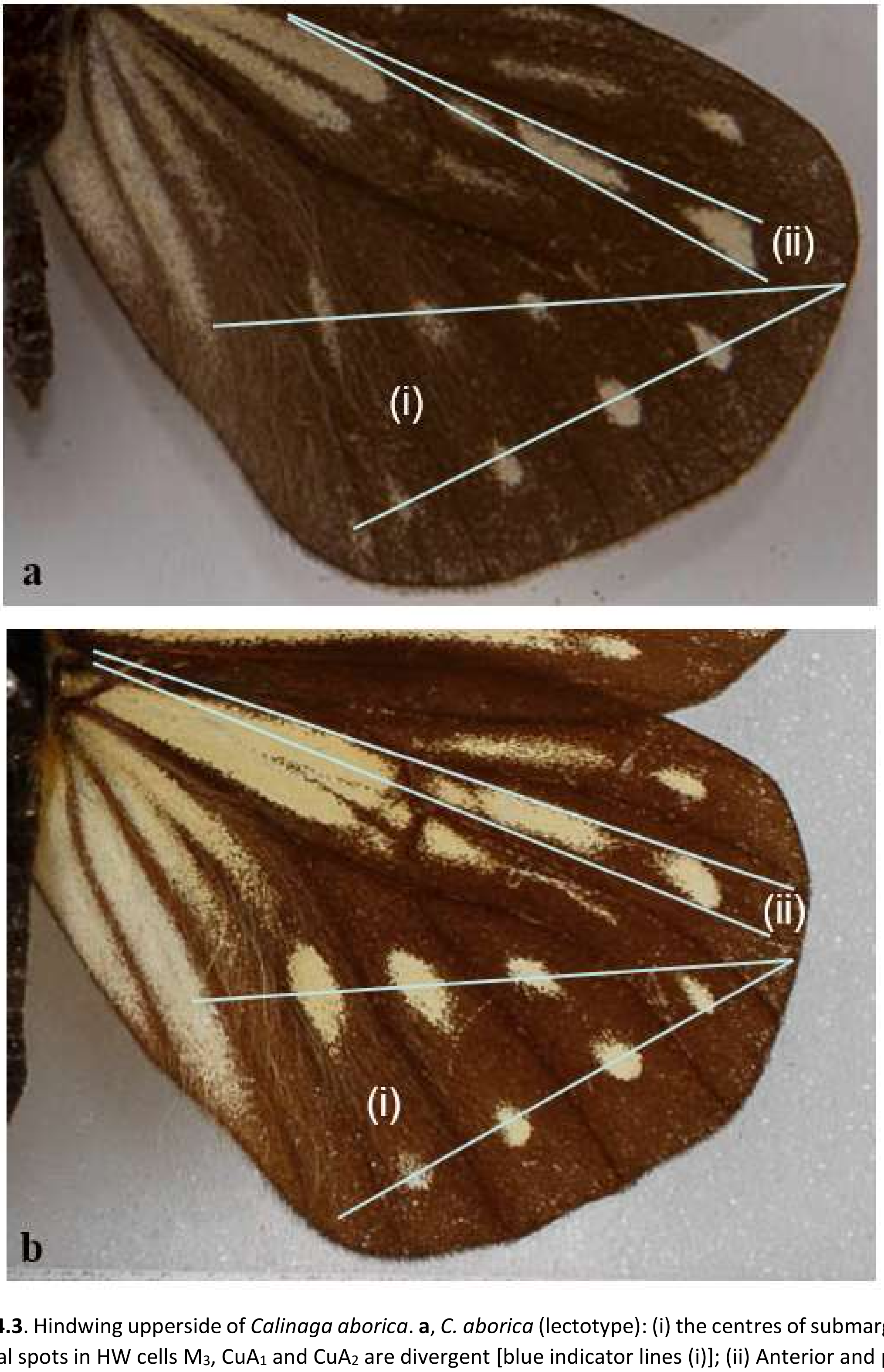

Figure S4.3. Hindwing upperside of *Calinaga aborica*. **a**, *C. aborica* (lectotype): (i) the centres of submarginal and postdiscal spots in HW cells M_3, CuA_1 and CuA_2 are divergent [blue indicator lines (i)]; (ii) Anterior and posterior tangential lines touching the main postdiscal streak and submarginal spot in HW cell M_1 converge well before the base of the wing [blue indicator lines (ii)]. **b**, *C. aborica naima* ssp. n. (paratype 010243161): (i) centres of submarginal and postdiscal spots in HW cells M_3, CuA_1 and CuA_2 divergent (as in Fig. a); (ii) Anterior and posterior tangential lines touching the main postdiscal streak and submarginal spot in HW cell M_1, even if converging, do not converge before the base of the wing (unlike Fig. a). [This figure also appears in the main text of Todisco *et*

al., 2024, as Fig. 6.]. ©The Trustees of the Natural History Museum, London (Creative Commons License 4.0 https://creativecommons.org/licenses/by/4.0/)

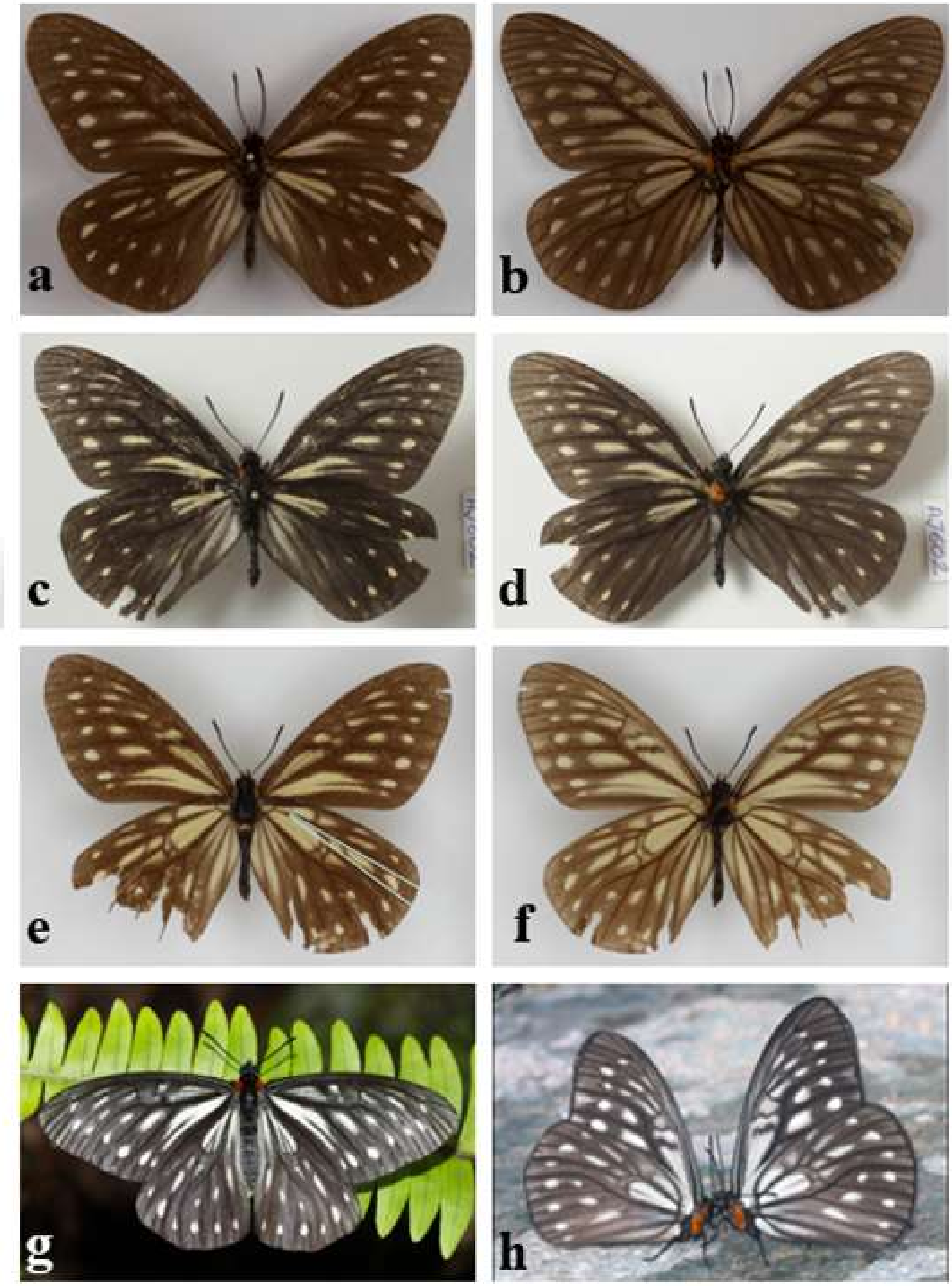

Figure S4.4. Male *Calinaga aborica aborica* (uppersides left, undersides right). **a,b**, lectotype, India, Abor Hills, June 1913, Porter leg., BMNH(E)#665228 (NHMUK, London); **c,d**, India, Arunachal Pradesh, East Kameng District, nr Sochung, Pakke Kesang, 21 May 2015, Sanjay Sondhi leg., NCBS-AJ602 (National Centre for Biological Sciences, Bengaluru); **e,f**, [Kabu], Yarlung Tsangpo river [Upper Brahmaputra], 6 June 1913, 3000 ft., F.M. Bailey (AMNH, New York) [tangential lines: cf Fig. S4.3a(ii)]; **g,h**, from Butterflies of India website, © Fahim Khan: **g**, India, Arunachal Pradesh, Upper Siang District, nr Yinkiong, 12 May 2023; **h**, same data except 18 May 2023. ©The Trustees of the Natural History Museum, London (Creative Commons License 4.0 https://creativecommons.org/licenses/by/4.0/).

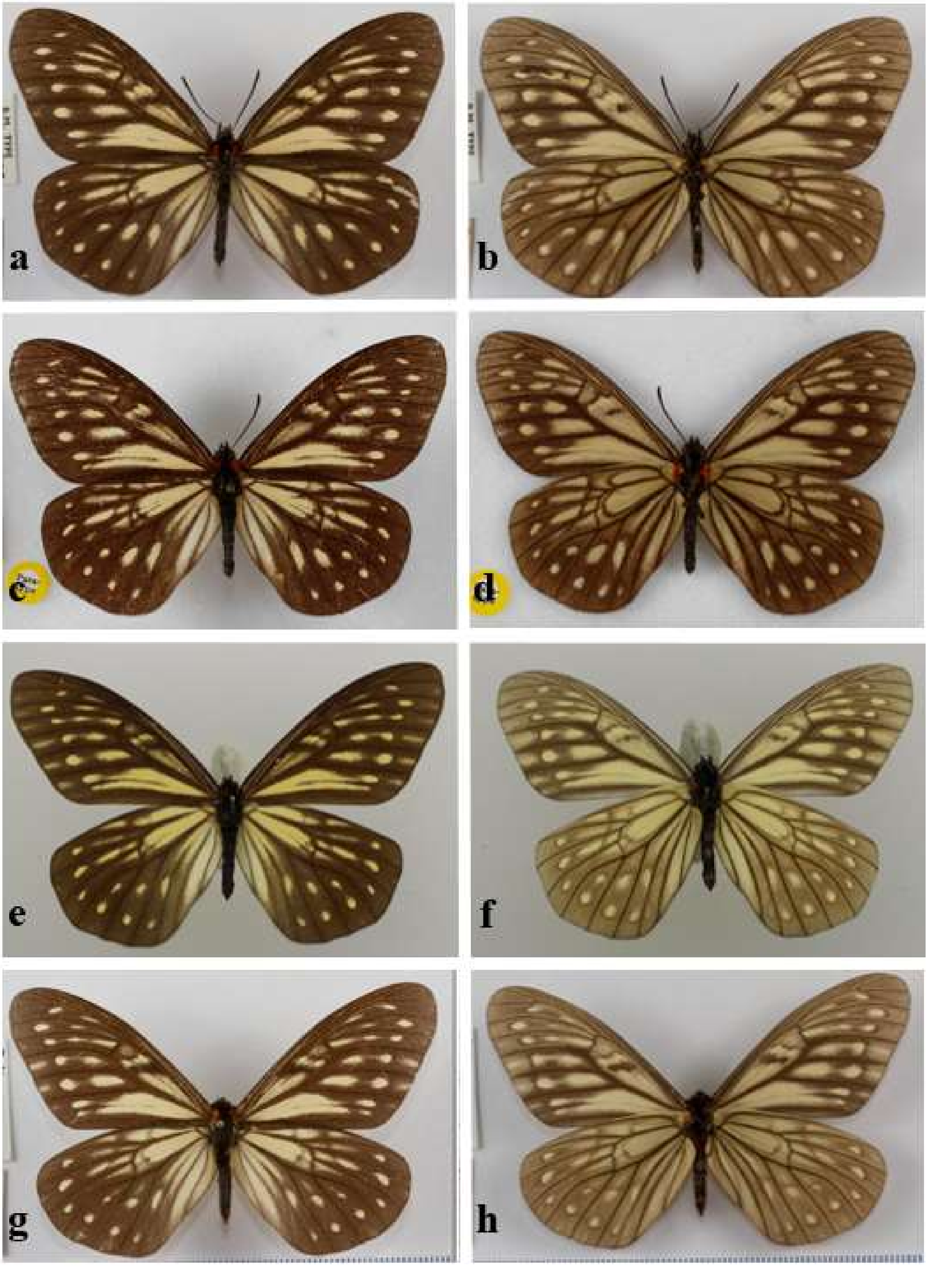

Figure S4.5. Male *Calinaga aborica naima* Vane-Wright (uppersides left, undersides right). **a,b**, holotype, Myanmar, Seinghku Valley, 6500′, 28.5 N 97.35 E., 27.v.1926, F. Kingdon Ward, BMNH(E)#985060 (NHMUK, London); **c,d**, paratype, Myanmar, Adung Valley, 6000′, 14 May 1931, Cranbrook, NHMUK010243281 (NHMUK, London); **e,f**, paratype, Myanmar, Kachin State, N.E. Putao. 5 May 1998, P. Sukkit leg. (Sukkit & Nishimura Collection); **g,h**, paratype, India, Arunachal Pradesh, Mishmi Hills, 10,000′, 13 June 1928, Percy Sladen Expedition, BMNH(E)#984922 (NHJMUK, London). [This figure, which also appears in the main text of Todisco *et al*. (2024), as Fig. 5, is repeated here to aid comparison with Fig. S4.4 (*C. aborica aborica*).] ©The Trustees of the

Natural History Museum, London (Creative Commons License 4.0 https://creativecommons.org/licenses/by/4.0/).

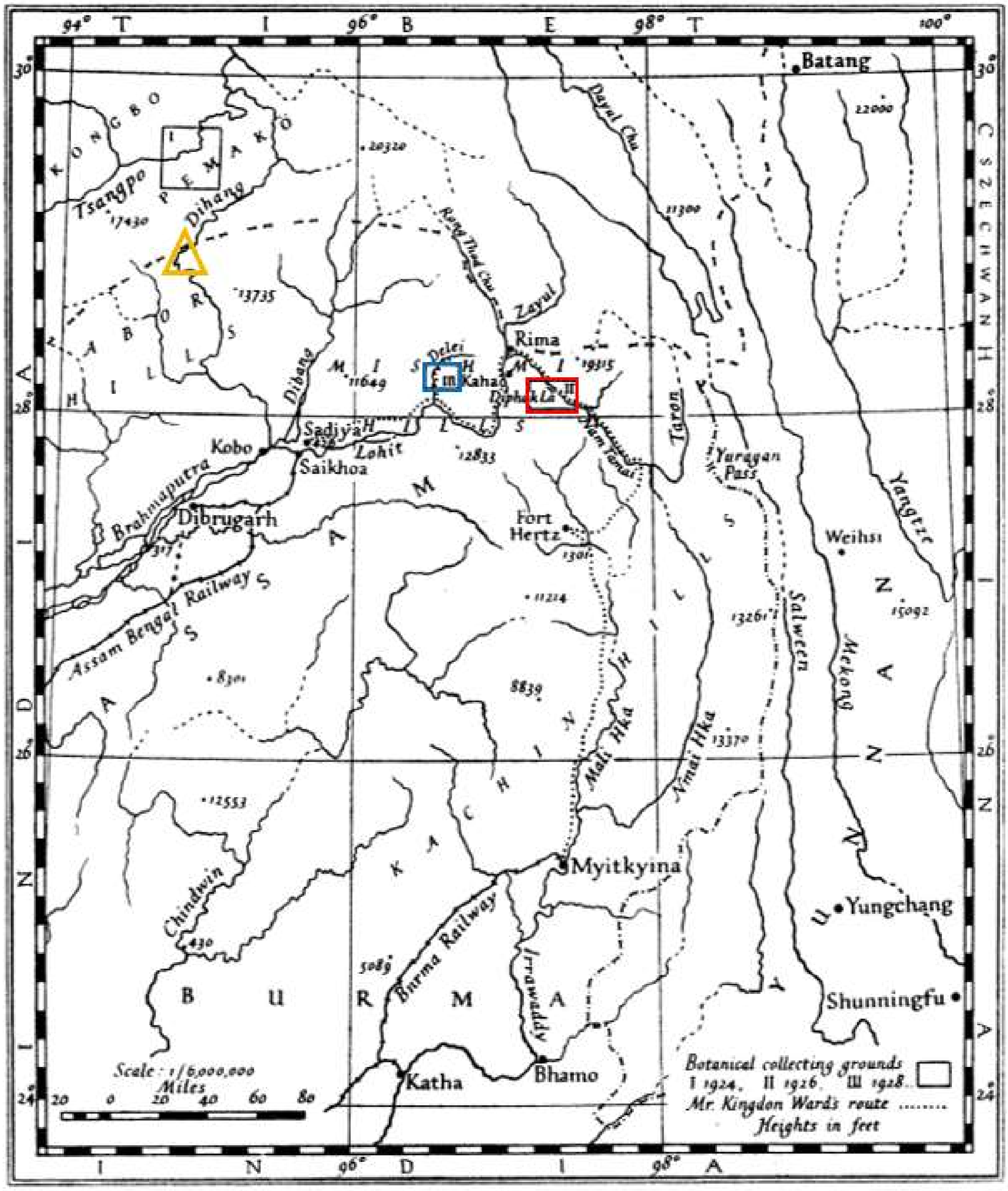

Figure S4.6. Map based on Kingdon Ward's (1930: fig. 1), 'Sketch-map of the Assam-Burma Border', with the approximate type locality for *Calinaga aborica* Tytler added (orange/yellow triangle), and where the holotype of *C. aborica naima*, ssp. nov. was collected by Kingdon-Ward (red rectangle). Note also the Delei Valley region (blue rectangle – see text). ©The Trustees of the Natural History Museum, London (Creative Commons License 4.0 https://creativecommons.org/licenses/by/4.0/).

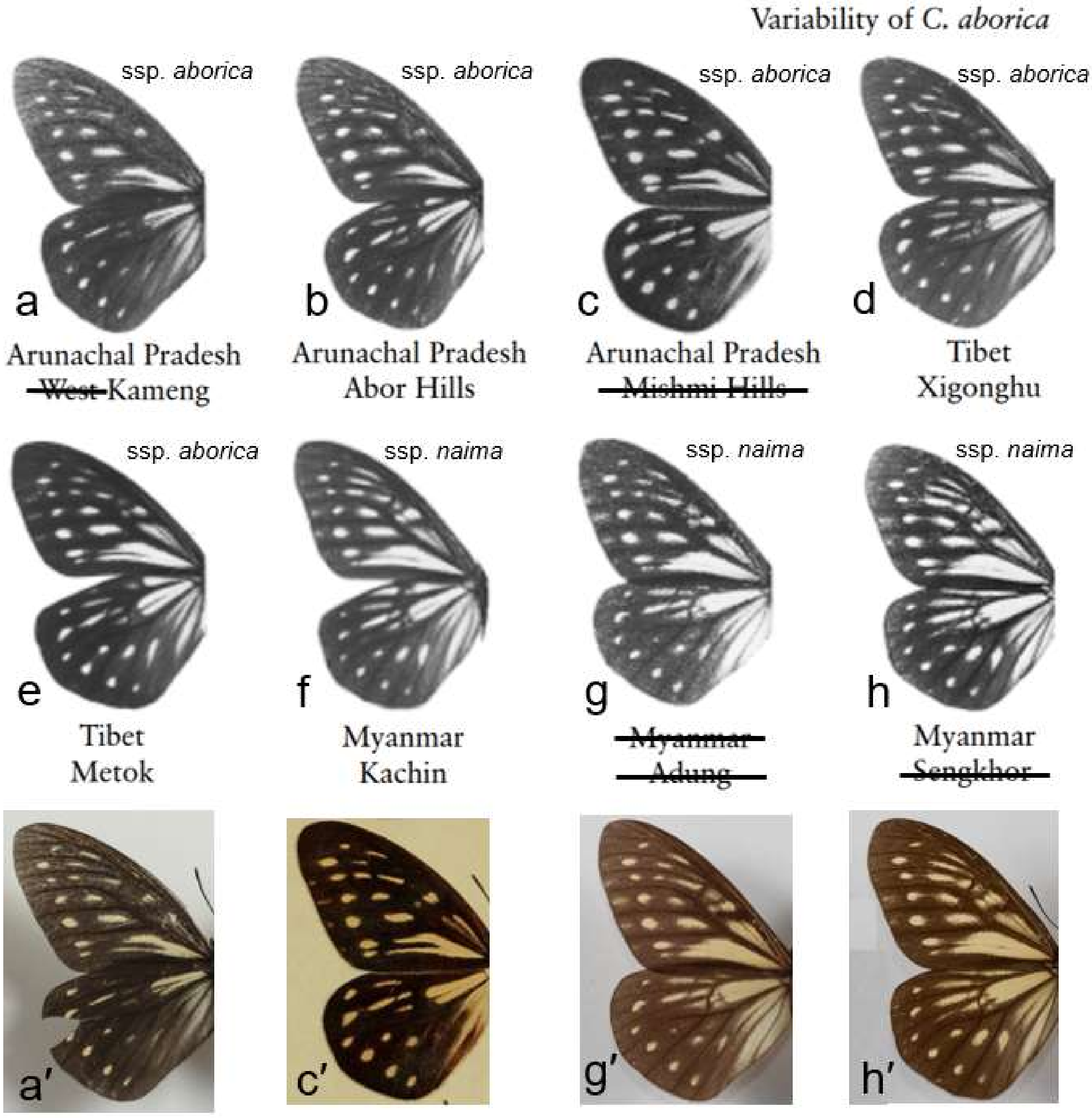

Figure S4.7. **a–h** “Variability of *C. aborica*”*,* based on a so-titled figure reproduced here from Tshikolovets (2020, fig. O, p. 78). Subspecies names, reference letters, strike-outs (indicating incorrect data), and the bottom row of figures, have been added. **a**,**a**′, both based on Sondhi *et al.* (2016, fig. C), which has the original data: Sochung, Pakke Kesang, East Kameng District, Arunachal Pradesh, India, 21 May 2015 [Fig. a appears to have been flipped 180° and ‘repaired’]; **b**, lectotype of *C. aborica*; **c**,**c**′, both based on the original illustration of what is now the paralectotype male *C. aborica,* from the Abor Hills (Tytler, 1915); **d**, based on Tshikolovets (2020 pl 26, fig. 3); **e**, based on Tshikolovets (2020 pl 26, fig. 5); **g**, **g**′, both based on paratype 9636 of *C. aborica naima*, from Arunachal Pradesh, Mishmi Hills; **h**,**h**′, both based on the holotype of *C. aborica naima*, with original data [Myanmar], Seinghku Valley. ©The Trustees of the Natural History Museum, London (Creative Commons License 4.0 https://creativecommons.org/licenses/by/4.0/).

REFERENCES

Ackery, P.R., de Jong, R. & Vane-Wright, R.I. (1999) The butterflies: Hedyloidea, Hesperioidea and Papilionoidea. In: Kristensen, N.P. (Ed.) *Lepidoptera, moths and butterflies, Volume 1: Evolution, Systematics, and Biogeography*. *Handbook of Zoology* 4(35). Berlin: Walter de Gruyter, pp. 263–300.

Anonymous. (2024) *Calinaga aborica* Tytler, 1915 – Dark Freak. In: Kunte, K., Sondhi, S. & Roy, P. (Eds) *Butterflies of India*, v. 4.12 [online]. Bangalore: Indian Foundation for Butterflies. https://www.ifoundbutterflies.org/calinaga-aborica (accessed 4.ii.2024.)

Bailey, F.M. (1914) Exploration on the Tsangpo or Upper Brahmaputra. *The Geographical Journal*, 44(4), 341–360, 1 map.

Cowan, C.F. (1975) Horsfield, Moore, and the catalogues of the East India Company Museum. *Journal of the Society for the Bibliography of Natural History*, 7(3), 273–284.

de Nicéville L. (1886) *The Butterflies of India, Burmah and Ceylon.* Calcutta: Calcutta Central Press.

de Nicéville L. (1900) Note on *Calinaga*, an aberrant genus of Asiatic butterflies. *Journal of the Asiatic Society of Bengal,* 69(2), 150–155.

Ehrlich, P.R. (1958) The comparative morphology, phylogeny and higher classification of the butterflies (Lepidoptera: Papilionoidea). *University of Kansas Science Bulletin*, 39(8), 305–370.

Ehrlich, P.R. (1959) A note on the systematic position of the butterfly genus *Calinaga* (Nymphalidae). *Lepidopterists' News*, 12, 173.

Freitas, A.V.L. & Brown Jr, K.S. (2004) Phylogeny of the Nymphalidae (Lepidoptera). *Systematic Biology*, 53(3), 363–383.

Hennig, W. (1966) *Phylogenetic systematics* (translated Davis, D.D. & Zangerl, R.). Urbana: University of Illinois Press.

Herrich-Schäffer, G.A.W. (1864) Prodromus systematis lepidopterorum. Versuch einer systematischen Anordnung der Schmetterlinge [part]. *Correspondenz-Blatt des zoologisch-mineralogischen Vereines in Regensburg*, 18(7/8), 89-112 (August); (9), 123-136 (September); (10), 148-152.

Jordan, K. (1898) Contributions to the morphology of Lepidoptera. *Novitates zoologicae*, 5(3), 374–415, 2 pls.

Kawahara, A.Y., Storer, C., Carvalho, A.P.S, Plotkin, D.M., Condamine, F.L., Braga, M.P., Ellis, E.A., and 80 other contributors. (2023) A global phylogeny of butterflies reveals their evolutionary history, ancestral hosts and biogeographic origins. *Nature Ecology & Evolution,* 7, 903–913. https://www.nature.com/articles/s41559-023-02041-9

Kingdon-Ward, F. (1930) The Seinghku and Delei valleys, north-east frontier of India. *The Geographical Journal*, 75(5), 412–432.

Kirby, W.F. (1871) *Synonymic catalogue of diurnal Lepidoptera*. London: JohnVan Voorst.

Klots, A.B. (1970) Lepidoptera. In: Tuxen, S.L. (Ed.), *Taxonomist's glossary of genitalia in insects* (2nd edn). Copenhagen: Munksgard, pp. 115–130.

Lambert, E.T.D. (1937) From the Brahmaputra to the Chindwin. *The Geographical Journal*, 89(4), 309–323.

Lang, S-Y. (2012) *The Nymphalidae of China (Lepidoptera, Rhopalocera). Part 1: Libytheinae, Danainae, Calinaginae, Morphinae, Heliconiinae, Nymphalinae, Charaxinae, Apaturinae, Cyrestinae, Biblidinae, Limenitinae* [sic]. Pardubice: Tshikolovets Publications.

Moore, F. [1858] In: Horsfield, T. & Moore, F. *A catalogue of the lepidopterous Insects in the museum of the Hon. East-India Company*. Volume I. London: W.H. Allen and Co. [For date of publication, see Cowan, 1975.]

Moore, F. (1895) *Lepidoptera Indica. Vol. II. Rhopalocera. Family Nymphalidae. Sub-families Satyrinae (continued), Elymniinae, Amathusiinae, Nymphalinae (group Charaxina).* London: L. Reeve & Co.

Morishita, T., Soe, H.M., Htay, H., Lwin, T.H., Guotana, J.M., Tamura, A., Mizukami, T. & Zaw, K. (2023) Origin and evolution of ultramafic rocks along the Sagaing Fault, Myanmar. *Journal of Earth Science*, 34(1), 122–132. https://doi.org/10.1007/s12583-021-1435-x

Salvi, D., Mathew, G., Kohn, B., Pande, K. & Borgohain, B. (2020) Thermochronological insights into the thermotectonic evolution of Mishmi Hills across the Dibang Valley, NE Himalayan Syntaxis. *Journal of Asian Earth Sciences*, 190, 104158.

Shizuya, H., Watanabe, Y. & Saito, M. (2011) Basic information on butterflies of Kachin state, Myanmar (part 5). *Butterflies (Teinopalpus)*, (59), 11–26.

Sondhi, S., Karmakar, T., Sondhi, Y., Jhaveri, R. & Kunte, K. (2016) Re-discovery of *Calinaga aborica* Tytler, 1915 (Lepidoptera: Nymphalidae: Calinaginae) from Arunachal Pradesh, India. *Journal of Threatened Taxa*, 8, 8618–8622. doi:10.11609/jott.2354.8.3.8618-8622

Thwin, H.K.M.M., Lwin, K.S., Renner, S.C. & Dumbacher, J.P. (2011) Ornithology of northern Myanmar. *Ornithological Monographs*, 70(1), 109–141. doi:10.1525/om.2011.70.1.109

Todisco, V., Huertas, B., Nath Basu, D., Kunte, K., Prosser, S.W.J., Russell, S., Mutanen, M., Zilli, A. & Vane-Wright, R.I. (2024). DNA barcodes from over-a-century-old type specimens shed light on the taxonomy of group of rare butterflies (Lepidoptera: Nymphalidae: Calinaginae). *PLOS ONE.*

Tshikolovets, V. (2020) *The genus* Calinaga *Moore (1858) (Lepidoptera: Nymphalidae, Calinaginae)*. Pardubice: Tshikolovets Publications.

Tytler, H.C. (1915) Notes on some new and interesting butterflies from Manipur and the Naga Hills. Part II. *Journal of the Bombay Natural History Society*, 23, 502–515, 4 pls. [Pl 3 was published in the same journal later in 1915, volume 24, between pages 118, 119.]

Vane-Wright, R.I. (2003) Evidence and identity in butterfly systematics. In: Boggs, C.L., Watt, W.B. & Ehrlich, P.R. (Eds.) *Butterflies: ecology and evolution taking flight*. Chicago: University of Chicago Press, pp. 477–513.

Vane-Wright, R.I. (2015) *Butterflies* (2nd edn). London: Natural History Museum.

Vane-Wright, R.I. (2020) *Euploea dorippus* Klug, 1845: species, semispecies, subspecies, junior subjective synonym of *Danaus chrysippus chrysippus* (Linnaeus), and/or form – what does Klug's *dorippus* represent? (Lepidoptera: Nymphalidae, Danainae). *Nota lepidopterologica*, 43, 117–138. doi 10.3897/nl.43.47936

Watson, J. (1899) On *Calinaga*, the single genus of an aberrant subfamily of butterflies. *Memoirs of the Literary and Philosophical Society of Manchester*, 43(11), 1–23, 3 pls.
