# Supplementary material for: DNA barcodes from over-a-century-old type specimens shed light on the taxonomy of a group of rare butterflies (Lepidoptera: Nymphalidae: Calinaginae)
Source: PLoS One. 2024 Jul 17;19(7):e0305825. doi: 10.1371/journal.pone.0305825 (PMC11253935; doi:10.1371/journal.pone.0305825)
Supplement: S4 File — (PDF) [file pone.0305825.s005.pdf]

SUPPLEMENTARY INFORMATION S5

Illustrated, Semi-Natural Key to the Major Subgroups of *Calinaga*

RICHARD I. VANE-WRIGHT

Scientific Associate, Insects Division, Department of Science, Natural History Museum, London SW7 5BD, UK

The current molecular tree, presented in the main body of this paper and revealing major clades within *Calinaga*, makes possible the construction of a semi-natural key to the species and species groups. This key is based entirely on the exo-phenotypic features of male adults, and is diagnostic wherever possible; it will probably also serve as an aid for identification of the very rarely encountered females (in some cases still unknown: e.g. *C. aborica*) – as long as it is remembered that females, on average, are significantly larger (as measured by forewing length); for the most part, their colour patterns are similar (but not identical) to their respective males.

Many of the best characters for identification are associated with the venation and coloration of the hind wings. *Calinaga* reveals the composite origin of cell CuA_2 in both wings, with the hindwings in particular having the course of resorbed posterior cubital vein (CuP) very strongly marked by dark scales (except at the extreme wing bases) – and this is so on both wing surfaces. While it is normal to refer to the entirety of the cell that lies between veins CuA_2 and 1A+2A as 'cell CuA_2', here it is convenient to refer to the anterior part only as cell CuA_2, and the posterior part as cell CuP. In accordance with the scheme adopted by Ackery & Vane-Wright (1984), the so called discocellular veins can be notated (in lower-case italics) by reference to the long veins that they connect (e.g. *r-m*, m_1-m_2, m_2-m_3, *m-cu*).

Two small departures from the current scheme of presented in the main text of Todisco *et al.* (2024) must be noted. The system of Tshikolovets (2020), who divided the genus into five superspecies (*buddha, brahma, formosana, davidis* and *lhatso*), once *C. aborica* is recognised as amply distinct from both the *davidis* complex and all other *Calinaga* (which is clearly evident on morphological grounds as well as molecular), then the major groups (putative clades) are similar in both schemes – with two exceptions, newly proposed by Todisco *et al.* These, I believe, might eventually prove to be as Tshikolovets suggested. Here I refer to the proposal to withdraw *C. lhatso senseiensis* Yoshino and *C. funeralis* Monastyrskii & Devyatkin from the *C. lhatso* group (subclade 1a in new scheme) and transfer them to subclade 1c (the *C. brahma* group). The reasons, based on colour pattern characters, can be summarised as follows:

The major pattern synapomorphy that links the three terminal taxa recognised by Tshikolovets that fall within subclade 1c (*brahma, sudassana, bedoci*) is the bright red hair that clothes the thoracic dorsum extending posteriorly, to include the metathorax – a feature not seen anywhere else in the genus, including *senseiensis* and *funeralis.*

The major pattern synapomorphy that links all six terminal taxa recognised by Tshikolovets as superspecies *lhatso* is the unique coloration of hindwing cell CuA_2 (not

including CuP – as above). In all members of this grouping (*pacifica, senseiensis, lhatso, funebris, funeralis,* '*dubernardi*'), the underside of this cell does not have a small, rounded, pale submarginal spot (or a similar postdiscal spot). On the upperside (with the exception of the problematic '*dubernardi*'), these taxa have the distal *ca* two-fifths of the cell space filled with pale yellow (proximally) and orange (distally), this scaling covering the full width of the cell. This arrangement is usually masked in *pacifica*, which typically has all the posterior cells of the hindwing upperside uniformly pale yellow; however, Tshikolovets (2020: pl. 35, figs 7, 9) illustrates two infuscated specimens of *pacifica* in which this same underlying pattern can just be discerned.

It could be argued that this arrangement is matched by many individuals of *C. brahma bedoci* and all *C. brahma sudassana* – i.e., those that have a more or less broad area of bright orange scaling at the tornus. However, these *brahma* forms never exhibit the two-tone effect (proximal yellow/distal orange) characteristic of the *lhatso* group; the orange coloration is subjectively different in the two groups; and about 50% individual *brahma*, *sudassana* and *bedoci* have the small, rounded submarginal spot in underside hindwing CuA_2 – a feature never seen in any individuals of the *lhatso* group (sensu Tshikolovets). In addition, there is a seemingly 'trivial' but unique feature of *senseiensis* that links it with both *pacifica* and *lhatso* (pale spots at the base of FW cells R_3 and R_4 – an arrangement not seen in any other *Calinaga* except some pale phenotypes within the *davidis* complex). Intriguingly, however, a peculiarity of *funeralis* that is matched in some individuals of *funebris* (a central, pale, tongue-like pattern element in the otherwise darker hindwing underside discal cell) also recurs, very occasionally, in *C. brahma*. Hybridization in the past might be involved.

There are features that can be used to group (*pacifica + senseiensis + lhatso*) on the one hand, and (*funebris + funeralis + yaonica*) on the other. We can call these groups L and D respectively (L = light; D = dark). Is it possible that L and D are not monophyletic? – it is difficult to assess the degree to which the red thoracic hair extends posteriorly in group D (there are no pictures of living dark-group taxa that I can find, and most museum specimens are rubbed) – but the type material of *funeralis* in NHMUK looks quite *brahma*-like in this regard. The two groups are easily segregated from each other by the postdiscal pale streak in FW cell CuA_2 upperside – in L group taxa this extends proximally to the fork of veins CuA_1 and CuA_2, often to the base of the wing; in the D group, this streak barely extends basally beyond the level of the fork. On this basis, '*dubernardi*' belongs within the L group.

In the key presented here, *senseiensis* and *funeralis* will key out as part of Clade 1a. Used in conjunction with the outstanding pictorial atlas of Tshikolovets (2020), this key should be of help with the identification of *Calinaga* butterflies, to the major groups.

Abbreviations used in the key: FW = forewing; HW = hindwing; ups = upperside (dorsal); uns = underside (ventral); fwl = forewing length.

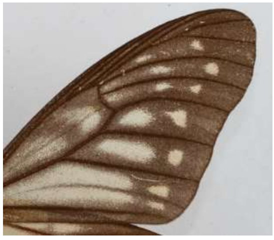

1. Larger species (fwl 40–60 mm); FW slightly but distinctly falcate (termen, or distal margin concave); HW uns cell CuA_2 with or without a small, submarginal pale spot occupying about one-third width of cell that contrasts with the darker ground colour; HW uns cell CuP only very rarely with a small submarginal spot (clade 1): **2**

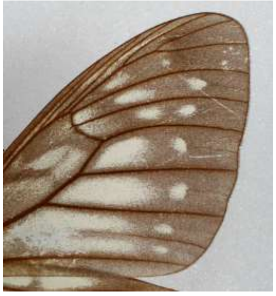

– Smaller species (fwl 33–50 mm); FW termen often rounded or straight (more rarely weakly concave at cell M3); HW uns cell CuA_2 usually with a small, submarginal pale spot and often a second, postdiscal such spot; HW uns cell CuP sometimes has one or two small spots: **5**

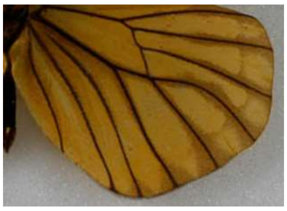

2 HW uns cell CuA_2 (and much of whole wing area) entirely yellow, without any discrete spots (clade 1a, part): ***Calinaga* (*lhatso*) *pacifica***

– HW uns cell CuA_2 not uni-colorous yellow, always with at least one spot or other variegation: **3**

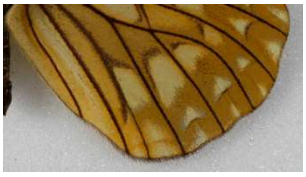

3 Outer half of HW uns cell CuA_2 with two or three wide, contrasting pattern elements, but never with a small rounded central spot (clade 1a, part): ***Calinaga* (*lhatso*) *lhatso*** complex

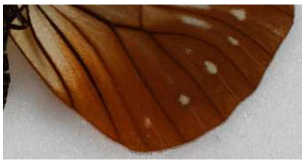

– HW uns cell CuA_2 not patterned as in *C. lhatso* complex; simply with or without a small, pale, contrasting submarginal spot; rarely a second small, postdiscal spot or long narrow streak: **4**

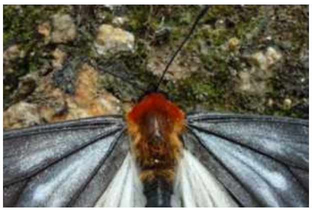

4 Bright red hairs of thoracic dorsum do not extend posterior of the mesothorax, the metathorax having golden hairs; underside HW ground colour pale yellowish-brown (clade 1b): ***Calinaga buddha*** complex

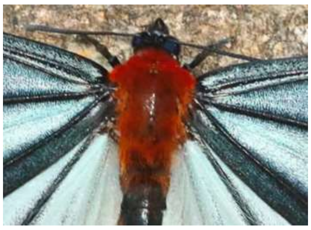

– Bright red hairs extend posteriorly to the metathorax; underside ground colour generally more chocolate-brown; some local populations have, on HW ups, a more or less extensive orange tornal area extending from cell CuA_2 to cell 2A (clade 1c):

Calinaga brahma complex

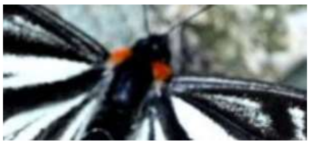

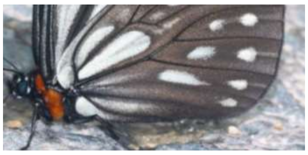

5 Red hairs on thoracic dorsum restricted to the tegulae, forming red ‘epaulettes’; HW cross veins m_1-m_2 and m_2-m_3 at a slight but distinct obtuse angle; m_2-m_3 meets M3 at junction with CuA_1; centres of submarginal and postdiscal spots in HW cell CuA_2 more widely separated than those in cell CuA_1, and the latter more widely separated than the corresponding spots in cell M_3 (clade 2): ***Calinaga aborica***

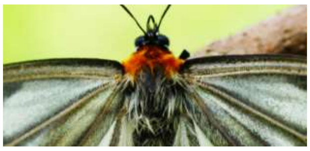

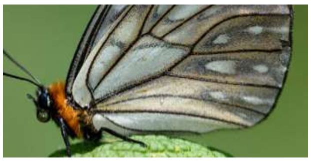

– Red or reddish hairs on thoracic dorsum not restricted to the tegulae, occurring across the width of the thorax; HW cross veins m_1-m_2 and m_2-m_3 rectilinear, or almost so; if both submarginal and postdiscal spots are present in HW cell CuA_2, their centres are not more widely separated than those in cell CuA_1, or those in cell M_3 : **6**

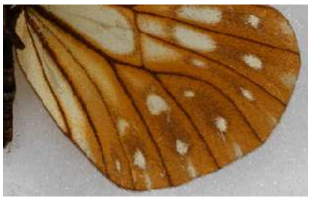

6 If both submarginal and postdiscal spots are present in HW cell CuA_2, their centres are significantly less widely separated than those of the corresponding spots in cell CuA_1; m_2-m_3 meets M_3 before, at, or just beyond its junction with CuA_1; vein CuA_2 straight or slightly bowed anteriorly; CuP almost invariably has a small submarginal spot; HW uns ground-colour pale reddish-brown(clade 3):

Calinaga formosana

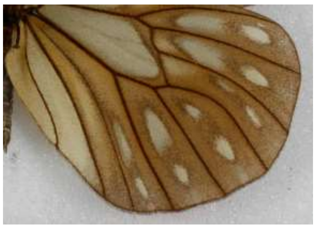

– If both submarginal and postdiscal spots are present in HW cell CuA_2, their centres are more or less equally separated compared to those of the corresponding spots in cell CuA_1 (and M_3); m_2-m_3 usually meets M_3 about 1–2 mm distal to the junction with CuA_1; vein CuA_2 at least slightly bowed posteriorly; CuP only occasionally has a small submarginal spot; HW uns ground-colour grey/yellowish-brown (clade 4): ***Calinaga davidis*** complex

Ackery, P.R. & Vane-Wright, R.I. (1984) *Milkweed butterflies*. Cornell: Cornell University Press.

Todisco, V., Huertas, B., Nath Basu, D., Kunte, K., Prosser, S.W.J., Russell, S., Mutanen, M., Zilli, A. & Vane-Wright, R.I. (2024). DNA barcodes from over-a-century-old type specimens shed light on the taxonomy of group of rare butterflies (Lepidoptera: Nymphalidae: Calinaginae). *PLOS ONE.*

Tshikolovets V (2020) *The genus* Calinaga *Moore, [1858] (Lepidoptera: Nymphalidae, Calinaginae).* Pardubice: Tshikolovets Publications.
