# Supplementary figures and images for: DNA barcodes from over-a-century-old type specimens shed light on the taxonomy of a group of rare butterflies (Lepidoptera: Nymphalidae: Calinaginae)
Source: PLoS One. 2024 Jul 17;19(7):e0305825. doi: 10.1371/journal.pone.0305825 (PMC11253935; doi:10.1371/journal.pone.0305825)

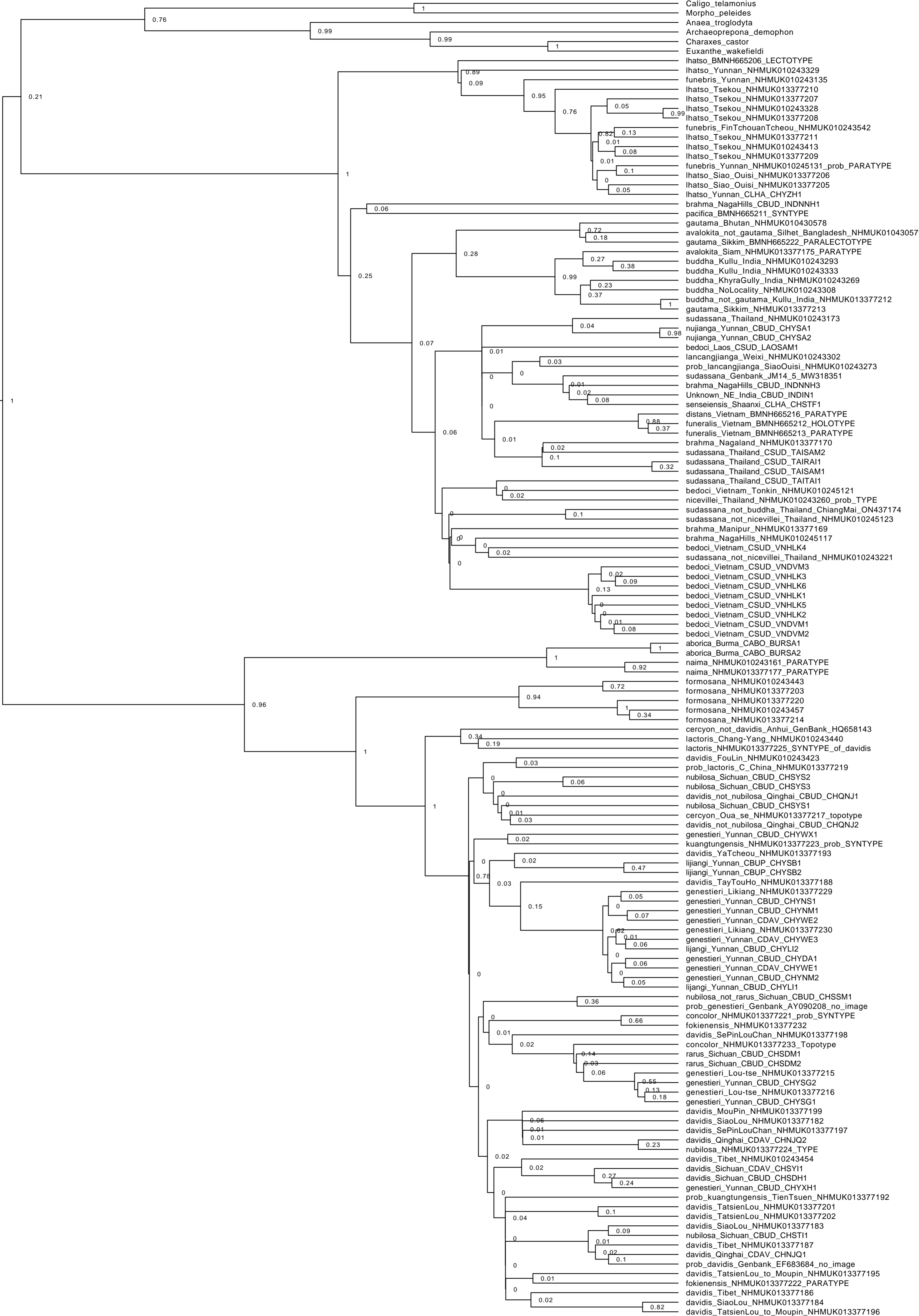

0.005

Supplement: S5 File — (PDF) [file pone.0305825.s006.pdf]
